# A *Ctnnb1* enhancer regulates neocortical neurogenesis by controlling the abundance of intermediate progenitors

**DOI:** 10.1038/s41421-022-00421-2

**Published:** 2022-08-02

**Authors:** Junbao Wang, Andi Wang, Kuan Tian, Xiaojiao Hua, Bo Zhang, Yue Zheng, Xiangfei Kong, Wei Li, Lichao Xu, Juan Wang, Zhiqiang Li, Ying Liu, Yan Zhou

**Affiliations:** 1 Department of Neurosurgery, Zhongnan Hospital of Wuhan University; Frontier Science Center for Immunology and Metabolism, Medical Research Institute at School of Medicine; The RNA Institute, College of Life Sciences; Wuhan University, Wuhan, Hubei China; 2 Department of Neurology, Wuhan Central Hospital, Tongji Medical College, Huazhong University of Science and Technology, Wuhan, Hubei China

**Keywords:** Neural stem cells, Neural stem cells

## Abstract

β-catenin-dependent canonical Wnt signaling plays a plethora of roles in neocortex (Ncx) development, but its function in regulating the abundance of intermediate progenitors (IPs) is elusive. Here we identified *neCtnnb1*, an evolutionarily conserved *cis*-regulatory element with typical enhancer features in developing Ncx. *neCtnnb1* locates 55 kilobase upstream of and spatially close to the promoter of *Ctnnb1*, the gene encoding β-catenin. CRISPR/Cas9-mediated activation or interference of the *neCtnnb1* locus enhanced or inhibited transcription of *Ctnnb1*. *neCtnnb1* drove transcription predominantly in the subventricular zone of developing Ncx. Knock-out of *neCtnnb1* in mice resulted in compromised expression of *Ctnnb1* and the Wnt reporter in developing Ncx. Importantly, knock-out of *neCtnnb1* lead to reduced production and transit-amplification of IPs, which subsequently generated fewer upper-layer Ncx projection neurons (PNs). In contrast, enhancing the canonical Wnt signaling by stabilizing β-catenin in *neCtnnb1*-active cells promoted the production of IPs and upper-layer Ncx PNs. ASH2L was identified as the key *trans*-acting factor that associates with *neCtnnb1* and *Ctnnb1*’s promoter to maintain *Ctnnb1*’s transcription in both mouse and human Ncx progenitors. These findings advance understanding of transcriptional regulation of *Ctnnb1*, and provide insights into mechanisms underlying Ncx expansion during development.

## Introduction

The expansion of the neocortex (Ncx), which in humans is the site of our cognitive abilities^1^, is thought to primarily be due to an increase in neuron production^2,3^. The excitatory/projection neurons (PNs) of the six-layered mammalian Ncx are born prenatally in an inside-out fashion. Briefly, Ncx radial glial cells (RGCs), which include basal RGCs and outer RGCs (oRGs), give rise to intermediate progenitors (IPs) residing in the subventricular zone. IPs can divide a few times (*a.k.a*. transit-amplification) before differentiating into PNs. In mice, deep-layer PNs are most generated between E11.5 to E14.5, followed by the production of upper-layer PNs, which migrate radially along the basal/radial fibers of RGCs and reside on top of deep-layer PNs^2,4–6^. IPs are morphologically and molecularly distinct from RGCs: IPs are multipolar and express markers including TBR2, NEUROG2 and BTG2. Notably, IPs first appear in amniotes, with some birds and reptiles having pallial structures similar to the mammalian Ncx^7,8^. Moreover, the Ncx expansion during development and evolution correlates with the pool size of oRGs and IPs^7,9–11^. Therefore, many recent studies have dissected cellular and molecular mechanisms underlying production, transit-amplification, and neuronal differentiation of neocortical IPs^12–14^.

The canonical Wnt signaling mediated by β-catenin is highly conserved through evolution and is essential for multiple aspects of brain development. It has been reported that the Wnt/β-catenin signaling promotes self-renewal of RGCs and neuronal differentiation of IPs, however, the role of Wnt/β-catenin signaling in production and properties of neocortical IPs remains inclusive, partly due to the involvement of β-catenin in cell cycle progressions, differentiation, and cell adhesion of neural cells^15–20^. Moreover, *CTNNB1* is one of the top risk genes associated with neurodevelopmental disorders including autism spectrum disorder (ASD)^21,22^. Much has been known regarding molecular components and signaling cascade for phosphorylation, degradation/stabilization, nuclear translocation of β-catenin^23,24^. However, transcriptional regulation of *Ctnnb1*, the gene that encodes β-catenin, is poorly understood^25^.

An enhancer is a *cis*-regulatory DNA element that spatiotemporally controls transcription of a particular gene in specific cell type(s). Many essential fate-specifying genes have multiple enhancers that are respectively responsible for its proper expression and function in distinct cell types^26,27^. An active enhancer is accessible by *tran*s-acting factors, enriched with chromatin signatures such as H3K27ac and H3K4me1, and spatially proximal to the promoter of its target gene^28,29^. Enhancer mutations and/or their aberrate activation/inactivation could lead to developmental abnormalities and cancers^26,30,31^. Thus, characterization of essential enhancers has implications in understanding molecular machineries that govern selective gene expression in developmental and disease scenarios.

Here we identified an evolutionarily conserved enhancer of *Ctnnb1*, the gene encodes β-catenin. The enhancer, *neCtnnb1*, exhibits specific activity in developing Ncx and is essential for production and transit-amplification of IPs and subsequent generations of upper-layer Ncx PNs. Finally, ASH2L was identified as the *trans*-acting factor that associates with *neCtnnb1* and *Ctnnb1*’s promoter to maintain *Ctnnb1*’s expression in both mouse and human Ncx progenitors.

## Results

### *neCtnnb1* is a conserved *cis*-regulatory element with typical enhancer features in developing neocortices

**Fig. 1:**
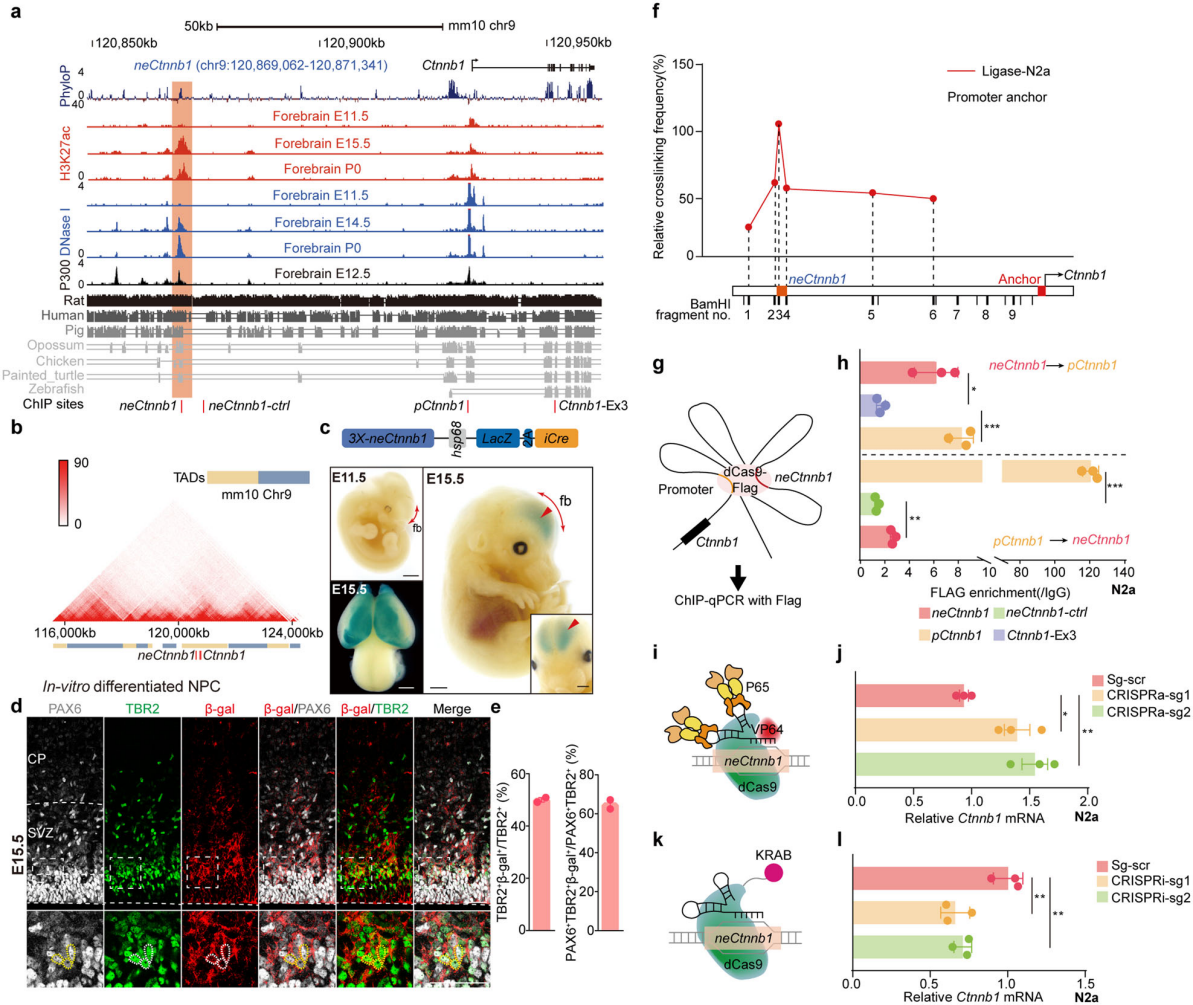
*neCtnnb1* is a neocortex-specific enhancer of *Ctnnb1*. **a** Schematic representation of the upstream region of mouse *Ctnnb1* gene and the location of putative enhancer *neCtnnb1* (2.28 kb, orange shading), which is evolutionarily conserved among amniotes and enriched with H3K27ac, DNase I hypersensitivity and P300 in developing forebrains. Data were obtained from ENCODE. Locations of ChIP sites in (**h**) were indicated. **b** Hi-C data of in-vitro differentiated NPCs were obtained from the 3D genome browser. Boundaries of the TADs and locations of *Ctnnb1* and *neCtnnb1* (red bar) were marked below. **c** Top: a schematic illustration showing the transgenic construct carries three tandem *neCtnnb1* sequences, the *hsp68* mini-promoter, the LacZ reporter gene and inducible CreERT2 (iCre). Bottom: X-Gal staining (blue) in E11.5 and E15.5 *neCtnnb1-LacZ-iCre* embryos and brains. Signals at E15.5 dorsal forebrains (fb) were indicated with red arrows and enlarged. **d** Immunofluorescence of PAX6 (gray), TBR2 (green), and β-Gal (red) on coronal sections of E15.5 *neCtnnb1-LacZ-iCre* Ncx. **e** Bar plots showing TBR2 + β-gal+ cells relative of TBR2 + cells (left) and PAX6 + TBR2 + β-gal+ cells relative of PAX6 + TBR2 + cells (right) in E15.5 *neCtnnb1-LacZ-iCre* Ncx. *n* = 2 for *neCtnnb1-LacZ-iCre* brains. Each point represents an individual brain. **f** The 3C assay performed using Neuro-2a neuroblastoma cells. The *Ctnnb1* promoter (*pCtnnb1*) is the anchor point from which long-range DNA interactions across the *neCtnnb1* interval were measured. Numbers over the data points represent fragment locations. **g** Schematics show the ChIP-qPCR evaluating the physical association between the *pCtnnb1* and *neCtnnb1*. **h** ChIP-qPCR measuring enrichment of *pCtnnb1* and *neCtnnb1* after Flag-tagged dCas9 were targeted to *neCtnnb1* and *pCtnnb1* respectively. Each point represents an independent experiment. **I**, **k** Schematics showing the CRISPR activation (CRISPRa, **i**) and CRISPR interference (CRISPRi, **k**) experiments. **j**, **l** RNA levels of *Ctnnb1* in Neuro-2a cells transfected with indicated CRISPRa (**j**) or CRISPRi (**l**) vectors for two days. In **h** quantification data are shown as means ± SEM, statistical significance was determined using two-way ANOVA followed by Tukey’s multiple comparisons test. In **j** and **l**, quantification data are shown as means ± SD, statistical significance was determined using one-way ANOVA analysis, *P < 0.05, **P < 0.01, and ***P < 0.001. ns, not significant. Scale bars, 1 mm (**c**), 50 μm (**d**). fb, forebrain; CP, cortical plate; VZ, ventricular zone; SVZ, subventricular zone.

We examined *cis*-regulatory elements that potentially regulate expressions of components of canonical Wnt signaling during Ncx development. We noticed that a genomic region — *neCtnnb1*, located 55 kilobase (kb) upstream of the transcription start site (TSS) of *Ctnnb1*, bears essential enhancer features, namely, prominent enrichment of DNase I hypersensitivity (HS), H3K4me1, H3K27ac, and P300 with low H3K4me3 and H3K36me3 signals in mid-late dorsal forebrains (shaded region in Fig. 1a, Supplementary Fig. S1a). The 2.28 kb genomic region of *neCtnnb1* (**n**eocortical **e**nhancer of *Ctnnb1*) contains a 696 bp evolutionarily conserved fragment and matches peaks of H3K27ac, DNase I HS and P300. However, these enhancer features are much less obvious in developing midbrains, hindbrains and in other non-neural tissues (Supplementary Fig. S1b–d). Moreover, analyses of Hi-C data revealed *neCtnnb1* and the gene body of *Ctnnb1* are in the same topologically associating domain (TAD) of in-vitro differentiated neural progenitor cells (NPCs) (Fig. 1b).

### *neCtnnb1* drives expression in intermediate progenitors (IPs) and newborn neurons of developing neocortices

Enhancers spatiotemporally control gene expression in the process of cell-fate determination during development^27,32,33^. We thus generated *neCtnnb1* transgenic mice, in which three 2.28-kb-long *neCtnnb1* sequences were tandemly engineered upstream of the *Hsp68* mini-promoter followed by a LacZ reporter and a 2A-CreERT2 cassette (*neCtnnb1-LacZ-iCre*) (Fig. 1c, top). Five F0 *neCtnnb1-LacZ-iCre* mouse founders were obtained and crossed with wild-type (WT) mice to study spatiotemporally expression patterns driven by *neCtnnb1*. 5-bromo-4-chloro-3-indolyl β-D-galactoside (X-Gal) staining of whole-mount and sectioned embryos of multiple lines consistently showed that *neCtnnb1* drove LacZ expression predominantly in dorsal forebrains at the peak of Ncx neurogenesis (Fig. 1c; Supplementary Fig. S2a–e), which corroborates with spatiotemporal distributions of epigenetic marks on *neCtnnb1*. Close-up observations found that at embryonic (E) day 15.5, the LacZ/β-Gal expression driven by *neCtnnb1* were mostly concentrated in the SVZ region of developing neocortices (Fig. 1d), with 50.0% of TBR2 + IPs express β-Gal (Fig. 1e). Particularly, immunofluorescent staining showed that *neCtnnb1*-driving β-Gal signal were detected in 64.8% of IP-differentiating RGCs that co-expressed PAX6 and TBR2 (Fig. 1d, e). Furthermore, the LacZ expression could be observed in cells co-labeled with TBR2 and NEUROD2 (Supplementary Fig. S2h), a marker for differentiating and mature PNs. Together, transgenic reporter mice showed that the enhancer activity of *neCtnnb1* is preferentially in embryonic SVZ, the predominantly neurogenic zone of the developing neocortex (Supplementary Fig. S2f, g).

### *neCtnnb1* associates with the promoter of *Ctnnb1* and maintains *Ctnnb1* expression

Enhancers usually have close proximity to promoter(s) of its target genes for transcriptional activation^34–36^. Setting *neCtnnb1* as the viewpoint, *neCtnnb1* was found to interact with *Ctnnb1*’s promoter (*pCtnnb1*) in differentiated NPCs and E14.5 cortical neurons using Hi-C and virtual 4C data (Supplementary Fig. S1e–h). Chromosome conformation capture (3C) experiments indeed revealed association of *neCtnnb1* with *pCtnnb1* in Neuro-2a neuroblastoma cells (Fig. 1f). We next carried out CRISPR/dCas9-mediated chromatin immunoprecipitation (ChIP) assay^27^. Data showed that the Flag-tagged dCas9 targeting *neCtnnb1* could significantly precipitate more *pCtnnb1* than the exon 3 of the *Ctnnb1* gene (*Ctnnb1*-Ex3). Consistently, Flag-tagged dCas9 targeting *pCtnnb1* could enrich more *neCtnnb1* than control locus (Fig. 1g, h). Moreover, CRISPR/dCas9-mediated activation (CRISPRa) or interference (CRISPRi) of the *neCtnnb1* locus greatly enhanced (Fig. 1i, j) or inhibited (Fig. 1k, l) *Ctnnb1*’s transcription. Thus, the long-range interaction between *neCtnnb1* and *pCtnnb1* might maintain *Ctnnb1*’s transcription in neural cells.

### *neCtnnb1* knock-out compromises the production of upper-layer (UL) PNs and *Ctnnb1* transcription

**Fig. 2:**
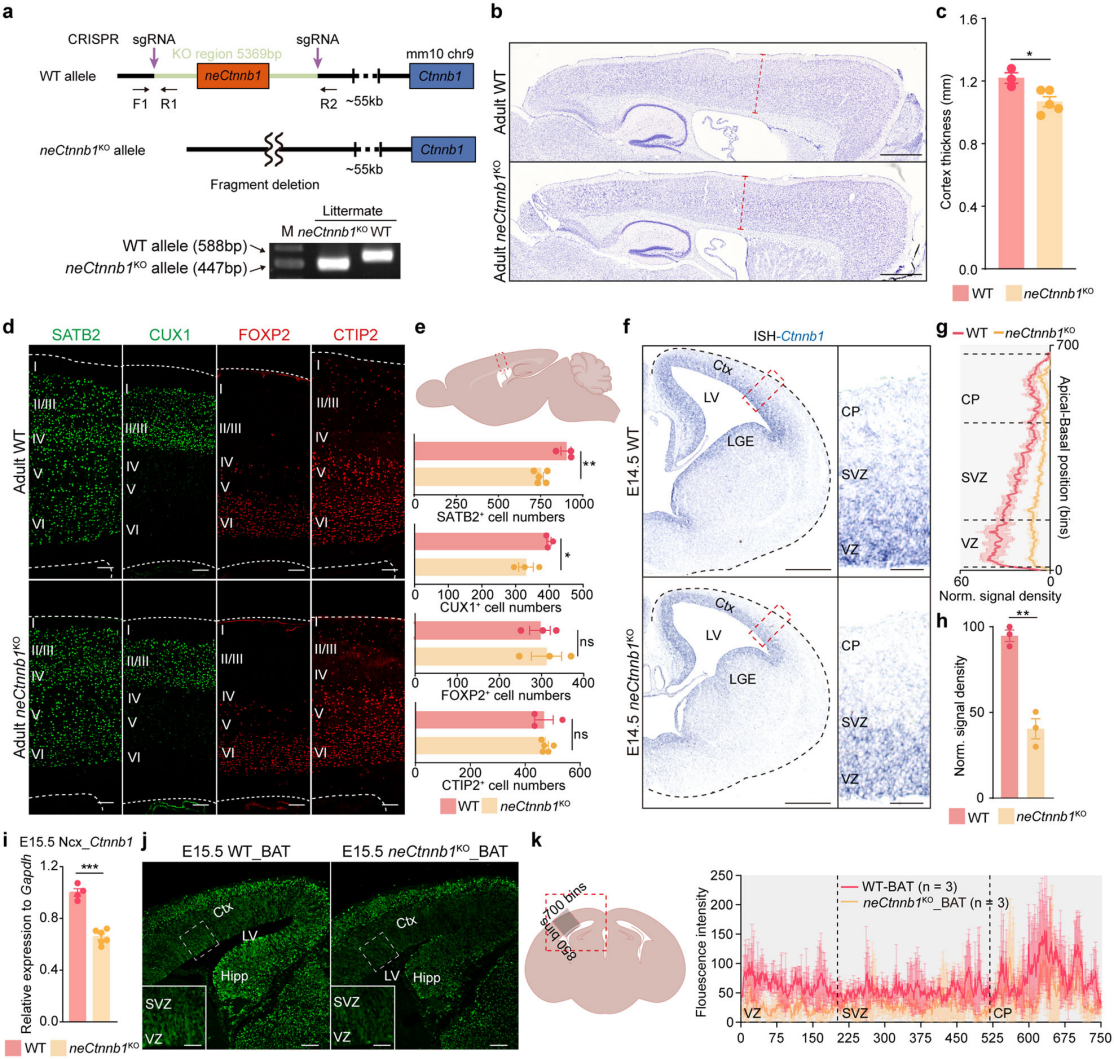
Knock-out of *neCtnnb1* in mice inhibits the production of upper-layer PNs and *Ctnnb1* transcription. **a** Generation and genotyping of *neCtnnb1*^KO^ mice. WT, wild-type; gRNA, guide RNA. **b** Nissl-staining on adult WT and *neCtnnb1*^KO^ sagittal brain sections. **c** Comparison of neocortical thickness of adult WT and *neCtnnb1*^KO^ mice. *n* = 3 for WT brains and *n* = 5 for *neCtnnb1*^KO^ brains. Each point represents an individual brain. **d**, **e** Immunofluorescence (**d**) and quantification (**e**, top) of SATB2 + , CUX1 + , FOXP2 + and CTIP2 + neurons on boxed area of sagittal sections (**e**) of adult WT and *neCtnnb1*^KO^ Ncx. Each point represents an individual brain. **f** In situ hybridization (ISH) of *Ctnnb1* on E14.5 WT (top) and *neCtnnb1*^KO^ (bottom) coronal brain sections, with boxed regions magnified on the right. **g**, **h** Quantification of normalized ISH signal densities in boxed regions of (**f**). *n* = 3 for WT brains and *n* = 3 for *neCtnnb1*^KO^ brains. Each point represents an individual brain. **i** RT-qPCR showing expressions of *Ctnnb1* in E15.5 WT and *neCtnnb1*^KO^ neocortex. *n* = 4 for WT Ncx and *n* = 6 for *neCtnnb1*^KO^ Ncx. Each point represents an individual brain. **j** β-Gal immunostaining on the E15.5 coronal sections of WT_BAT (left) and *neCtnnb1*^KO^_BAT (right) Ncx. Boxed regions are enlarged at the bottom left corners. **k** Quantification of normalized signal density of β-Gal in boxed regions of (**j**). *n* = 3 for WT brains and *n* = 3 for *neCtnnb1*^KO^ brains. Each point represents an individual brain. Quantification data are shown as means ± SEM. Statistical significance was determined using an unpaired two-tailed Student’s *t*-test (**c**, **e**, **h** and **i**). *P < 0.05, **P < 0.01, and ***P < 0.001. ns, not significant. Scale bars, 500 μm (**f**), 200 μm (**b**), 100 μm (**d** and **j**), 50 μm (magnified views in **f** and **j**). CP, cortical plate; VZ, ventricular zone; SVZ, subventricular zone; Ctx, cortex; LV, lateral ventricular; LGE, lateral ganglionic eminences; Hipp, Hippocampal primordium.

The canonical Wnt signaling mediated by nuclear-translocated β-catenin is essential for multiple aspects of Ncx development, including forebrain patterning^37,38^, area expansion^15,18^, self-renewal of radial glial cells (RGCs)^39,40^, and production of layer-specific neurons^16,41^. In addition, because β-catenin is the key component of apical adherens junctions, loss of β-catenin causes disorganized neuroepithelium^15,42^. To ask if *neCtnnb1* regulates Ncx development *via* controlling expression of β-catenin, we generated *neCtnnb1* knockout mice (*neCtnnb1*^KO^) wherein a 5,369-bp-long genomic region encompassing *neCtnnb1* was ablated using CRISPR/Cas9-mediated gene editing (Fig. 2a).

Homozygous *neCtnnb1*^KO^ mice were born according to Mendelian ratio with no visible defects (Supplementary Fig. S3a). Adult *neCtnnb1*^KO^ brains have the same Ncx hemisphere area and rostral-caudal length as WT controls (Supplementary Fig. S3b, c). However, the Ncx of adult *neCtnnb1*^KO^ brains were 10.8% thinner than WT controls (Fig. 2b, c). Immunofluorescent (IF) staining revealed that *neCtnnb1*^KO^ neocortices contained 14.2% and 17.1% fewer SATB2 + and CUX1 + UL PNs respectively with unaltered CTIP2 + and FOXP2 + deep-layer PNs (Fig. 2d, e), suggesting compromised neurogenesis during mid-late embryonic development.

We next examined whether expressions of *Ctnnb1* and transcriptional targets of canonical Wnt signaling were compromised in *neCtnnb1*^KO^ Ncx, particularly during cortical neurogenesis. In situ hybridization (ISH) of WT E14.5 Ncx showed an apical high to basal low gradient of *Ctnnb1* expression (Fig. 2f). Strikingly, the *Ctnnb1* expression was greatly compromised throughout the *neCtnnb1*^KO^ Ncx (Fig. 2g, h). Reverse transcription-quantitative PCR (RT-qPCR) studies further confirmed that expressions of *Ctnnb1* were greatly reduced in E15.5 *neCtnnb1*^KO^ Ncx (Fig. 2i). Consistently, the BAT-Gal reporter mice indicated that ablation of *neCtnnb1* lead to reduced Wnt-reporter expression, reflected by β-galactosidase IF signals (Fig. 2j, k) and the LacZ staining, throughout E15.5 and postnatal (P) day 8 Ncx (Supplementary Fig. S3d–j).

### *neCtnnb1* knockout hampered the production and transit-amplification of IPs

**Fig. 3:**
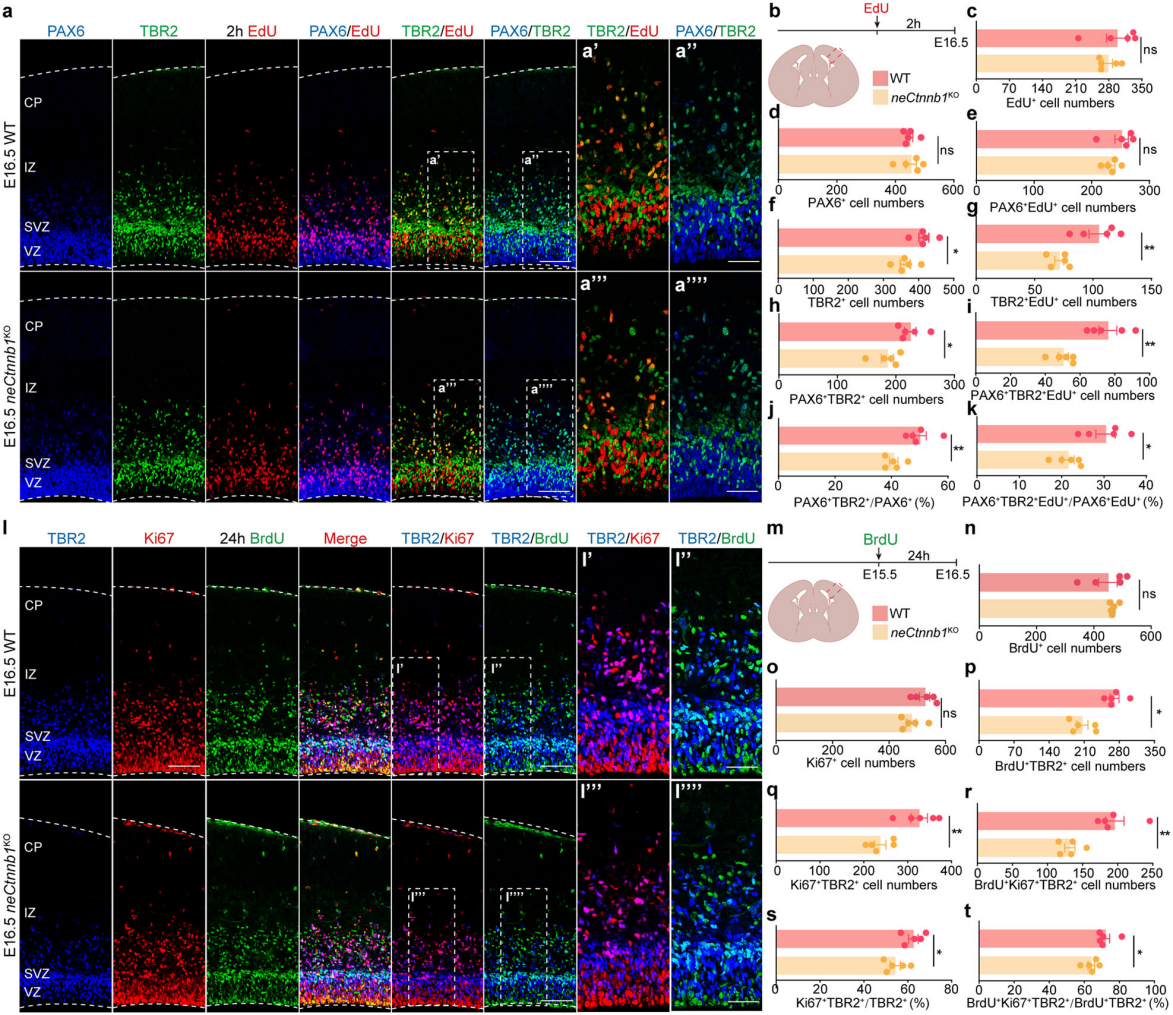
*neCtnnb1* knockout hampered the production and transit-amplification of IPs. **a**–**k** Immunofluorescence (**a**) and quantification (**c**–**k**) of EdU + (red), PAX6 + (blue) and TBR2 + (green) cells on boxed area of coronal sections (**b**) of E16.5 WT and *neCtnnb1*^KO^ Ncx. Pregnant mice were injected with EdU 2 h before sacrifice. Each point represents an individual brain. **l**–**t** Immunofluorescence (**l**) and quantification (**n**–**t**) of Ki67 + (red), TBR2 + (blue) and BrdU + (green) cells on boxed area of coronal sections (**m**) of E16.5 WT and *neCtnnb1*^KO^ Ncx. Pregnant mice were injected with BrdU at E15.5. **m**–**t** Quantification of data in **l**. Each point represents an individual brain. Quantification data are shown as means ± SEM. Statistical significance was determined using an unpaired two-tailed Student’s *t*-test (**c**–**k**, **n**–**t**). *P < 0.05, **P < 0.01, and ***P < 0.001. ns not significant. Scale bars, 100 μm (**a** and **l**), 50 μm (magnified views in **a**, **l**).

We then asked whether the compromised neurogenesis of UL PNs in *neCtnnb1*^KO^ cortices was due to alterations of Ncx progenitor pool and/or their behaviors. E16.5 *neCtnnb1*^KO^ and control embryos were pulse-labeled with 2 h EdU and subjected to IF analyses (Fig. 3a, b). First, although numbers of PAX6 + RGCs and total EdU+ cells were not significantly altered in E16.5 *neCtnnb1*^KO^ Ncx (Fig. 3c, e), the number of TBR2+ IPs was reduced by 12.7% (Fig. 3f), indicating compromised production of IPs by RGCs and/or hampered transit-amplification of IPs. Indeed, numbers of PAX6 + TBR2 + double-positive cells and the ratio of PAX6 + TBR2 + among all PAX6 + cells were decreased by 17.4% and 18.5% respectively in *neCtnnb1*^KO^ Ncx (Fig. 3h–j), pointing to compromised production of IPs by RGCs upon ablation of *neCtnnb1*. Moreover, although the number of RGCs at the proliferation status (PAX6 + EdU +) were unchanged upon loss of *neCtnnb1* (Fig. 3e), the dividing capacity of *neCtnnb1*^KO^ IPs was greatly compromised, as 32.1% fewer or 30.8% less TBR2 + IPs were co-localized with EdU (Fig. 3g) or 0.5 h bromodeoxyuridine (BrdU) (Supplementary Fig. S4a, b) respectively in *neCtnnb1*^KO^ neocortices. In addition, the *neCtnnb1*^KO^ Ncx contained 33.7% fewer PAX6 + TBR2 + EdU+ cells and 29.0% less PAX6 + TBR2 + EdU+ cells among PAX6 + EdU+ cells, further indicating compromised IP production from RGCs upon loss of *neCtnnb1* (Fig. 3i–k).

Next, embryos were pulse-labeled by BrdU 24 h prior to analyses at E16.5 by triple-labeling of BrdU, Ki67 and TBR2 (Fig. 3l, m). Numbers of cells labeled by 24 h BrdU or Ki67 were unchanged in *neCtnnb1*^KO^ Ncx (Fig. 3n, o). In contrast, 23.8% fewer TBR2 + cells in *neCtnnb1*^KO^ Ncx were labeled by 24 h BrdU (Fig. 3p), further supporting diminished production of IPs upon loss of *neCtnnb1*. Moreover, significantly fewer TBR2 + IPs were actively dividing at E16.5 (27.3% fewer Ki67 + TBR2 + cells and 13.2% less Ki67 + TBR2 + /TBR2 + cells, Fig. 3q, s), and stayed in cell cycle during the 24 h period (32.7% fewer BrdU+Ki67 + TBR2 + cells and 11.1% less BrdU+Ki67 + TBR2 + /BrdU+TBR2 + cells, Fig. 3r, t) in *neCtnnb1*^KO^ Ncx. Collectively, the production and transit-amplification/divisions of neocortical IPs were greatly compromised upon loss of *neCtnnb1*.

A previous study demonstrated that transient downregulation of canonical Wnt/β-catenin signaling plays a critical permissive role for proper polarization and radial migration of Ncx PNs^43^, which prompted us to examine whether deletion of *neCtnnb1* would hamper neuronal migration. To this end, constructs that express EGFP were electroporated into E14.5 neocortices and Ncx sections were immunostained with SATB2 at E18.5 (Supplementary Fig. S4c, d). In *neCtnnb1*^KO^ Ncx, the destination and ratio of SATB2 expression of electroporated cells and their progeny labeled by EGFP were the same pattern as those in the WT Ncx (Supplementary Fig. S4e, f), suggesting deletion of *neCtnnb1* has no effect on neuronal migration.

Of note, the ventricle surface of *neCtnnb1*^KO^ Ncx is as smooth and intact as WT Ncx without ectopic neurogenic foci (Fig. 2b, f), indicating that decreased *Ctnnb1* expression throughout Ncx caused by *neCtnnb1*^KO^ has no effect on cellular architecture of developing Ncx.

### Stabilization of β-catenin in *neCtnnb1*-active cells promotes the production and transit-amplification of IPs

**Fig. 4:**
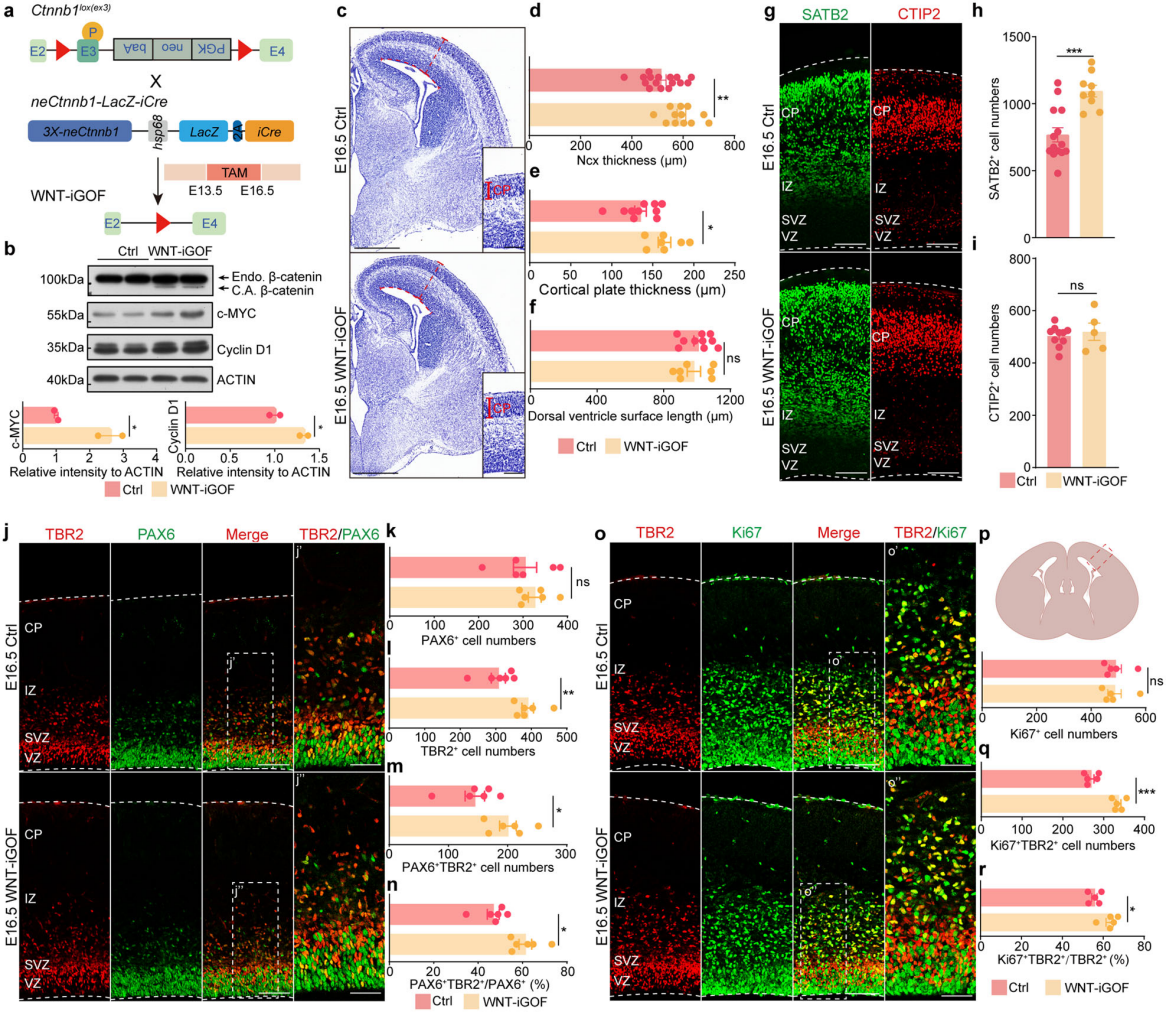
Stabilization of β-catenin in *neCtnnb1*-active cells promotes the production and transit-amplification of IPs in developing neocortices. **a** Schematics show mating of *Ctnnb1*^*lox(ex3)*^ mice with *neCtnnb1-LacZ-iCre* mice. Pregnant mice were injected with tamoxifen (TAM) at E13.5 and sacrificed at E16.5. **b** Top: immunoblotting of β-catenin, c-MYC, Cyclin D1 and ACTIN using protein lysates of Ctrl - *Ctnnb1*^*lox(ex3)*^ and WNT-iGOF - *neCtnnb1-LacZ-iCre*;*Ctnnb1*^*lox(ex3)*^ neocortices. Bottom: histograms show protein levels of c-MYC and Cyclin D1 normalized to ACTIN levels. Values of Ctrl neocortices were set as ‘1’. C.A. β-catenin, the constitutively active (non-degradable) β-catenin caused by deletion of *Ctnnb1*’s exon 3; Endo. β-catenin, endogenous β-catenin. **c** Nissl-staining on E16.5 Ctrl and WNT-iGOF coronal brain sections. **d** Comparison of neocortical thickness of E16.5 Ctrl and WNT-iGOF mice. *n* = 16 for Ctrl brains and *n* = 12 for WNT-iGOF brains. Each point represents an individual brain. **e** Comparison of cortical plate thickness of E16.5 Ctrl and WNT-iGOF mice. *n* = 10 for Ctrl brains and *n* = 7 for WNT-iGOF brains. Each point represents an individual brain. **f** Comparison of dorsal ventricle surface length of E16.5 Ctrl and WNT-iGOF mice. *n* = 10 for Ctrl brains and *n* = 7 for WNT-iGOF brains. Each point represents an individual brain. **g**–**i** Immunofluorescence (**g**) and quantification (**h**, **i**) of SATB2 + and CTIP2 + of coronal sections of E16.5 Ctrl and WNT-iGOF Ncx. **j**–**n** Immunofluorescence (**j**) and quantification (**k**–**n**) of of TBR2 + (red) and PAX6 + (green) cells on boxed area of E16.5 coronal sections (**p**) of Ctrl and WNT-iGOF Ncx. *n* = 6 for Ctrl brains and *n* = 6 for WNT-iGOF brains. Each point represents an individual brain. **o**–**r** Immunofluorescence (**o**) and quantification (**p**–**r**) of TBR2 + (red) and Ki67 + (green) cells on boxed area of coronal sections (**p**) of E16.5 Ctrl and WNT-iGOF Ncx. *n* = 5 for Ctrl brains and *n* = 5 for WNT-iGOF brains. Each point represents an individual brain. Quantification data are shown as means ± SEM. Statistical significance was determined using an unpaired two-tailed Student’s *t*-test (**d**, **e**, **f**, **h**, **i**, **k**–**n** and **p**–**r**). *P < 0.05, **P < 0.01, and ***P < 0.001. ns not significant. Scale bars, 500 μm (**c**), 100 μm (**g**, **j**, **o** and magnified views in **c**). 50 μm (magnified views in **j** and **o**).

We next explored whether activating the canonical Wnt/β-catenin signaling in *neCtnnb1*-active cells could promote the production and transit-amplification of IPs, hence enhance Ncx neurogenesis. *neCtnnb1-LacZ-iCre* mice were crossed with conditional *Ctnnb1*^*lox(ex3)*^ mice, with tamoxifen (TAM) injected at E13.5 and embryos collected at E16.5 (Fig. 4a). As expected, the non-degradable constitutively-active β-catenin (C.A. β-catenin) could be detected in E16.5 *Ctnnb1*^*lox(ex3)*^;*neCtnnb1-LacZ-iCre* (WNT-iGOF) Ncx, and products of Wnt/β-catenin target genes including *C-Myc* and *Cyclin D1* (*Ccnd1*) were also significantly enhanced in the WNT-iGOF Ncx (Fig. 4b). Strikingly, the E16.5 WNT-iGOF Ncx and their cortical plates were thicker than *Ctnnb1*^*lox(ex3)*^ controls (Fig. 4c, e; Supplementary Fig. S4g–i), with 42.6% more SATB2 + UL PNs and 26.9% more TBR2 + IPs in the E16.5 WNT-iGOF Ncx (Fig. 4h, l). But the numbers of PAX6 + RGCs and CTIP2 + deep-layer PNs were not changed in the WNT-iGOF Ncx (Fig. 4i, k). The length of dorsal ventricle surfaces was not expanded in the WNT-iGOF Ncx (Fig. 4f; Supplementary Fig. S4j), suggesting that activation of the canonical Wnt/β-catenin signaling by *neCtnnb1* did not cause lateral expansion of RGCs and Ncx, which could be otherwise observed in other Wnt/β-catenin gain-of-function studies^18,40^. Importantly, the number of PAX6 + TBR2 + double-positive cells and ratio of PAX6 + TBR2 + among all PAX6 + cells were increased by 38.7% and 30.8% respectively in E16.5 WNT-iGOF Ncx (Fig. 4j, m, n), indicative of enhanced production of IPs by RGCs. In addition, 25% more TBR2 + IPs were actively cycling and expressed Ki67, and the ratio of IPs that expressed Ki67+ increased by 13.4% in WNT-iGOF Ncx (Fig. 4o–r). Consistently, 37.8% more TBR2 + IPs were labeled with 0.5 h BrdU, and a larger portion of 0.5 h BrdU+ cells were IPs in WNT-iGOF Ncx (Supplementary Fig. S4k–n). Again, the integrity of epithelial structure was not altered in WNT-iGOF Ncx, reinforcing the notion that *neCtnnb1* controls Ncx neurogenesis independent of the cell-adhesion function of β-catenin (Fig. 4c; Supplementary Fig. S4g). Together, *neCtnnb1*-mediated canonical Wnt signaling is required and sufficient for generation and transit-amplification of IPs, hence proper production of UL PNs.

### ASH2L associates with *neCtnnb1* and *pCtnnb1* to regulate *Ctnnb1*’s transcription in mouse and human Ncx progenitors

**Fig. 5:**
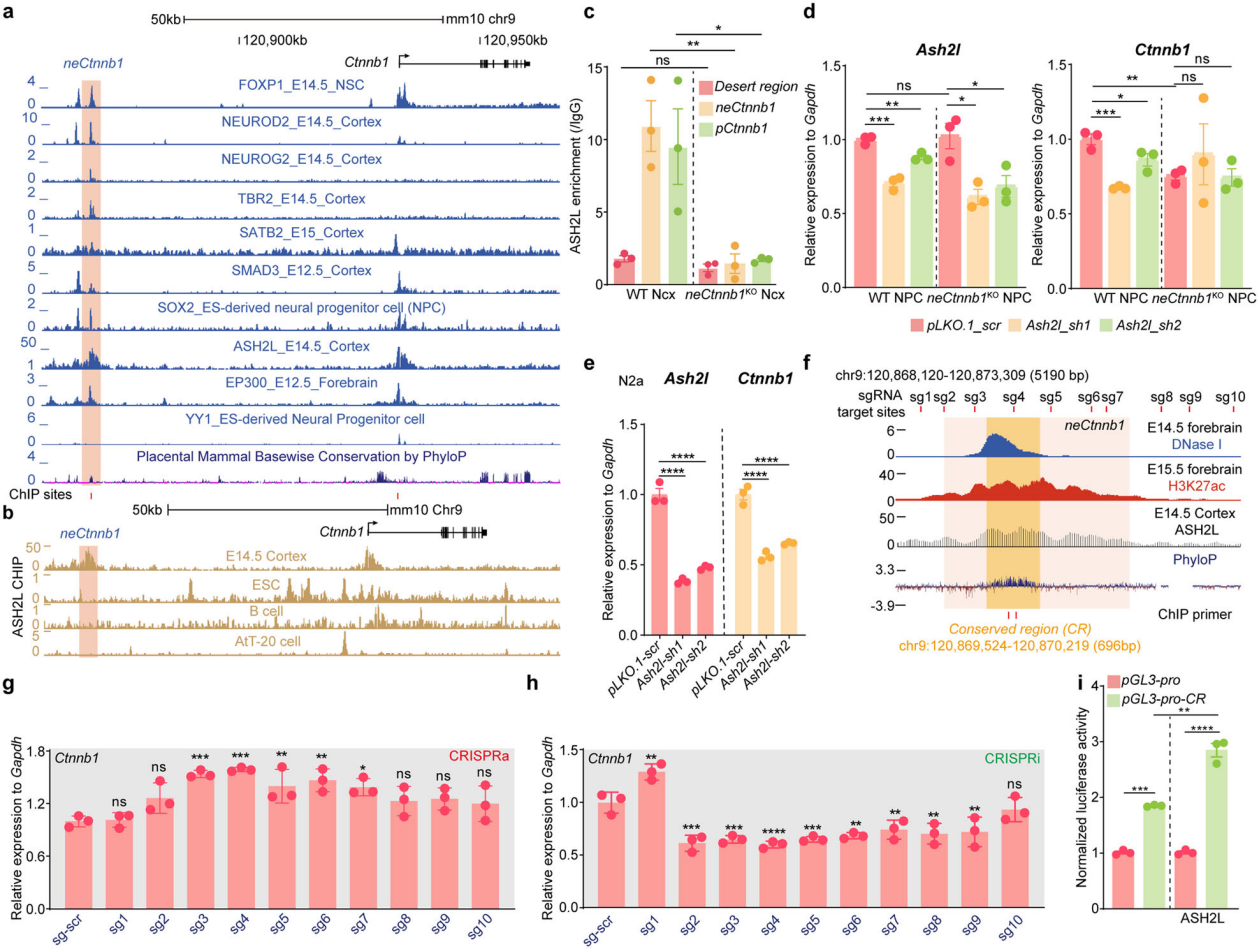
ASH2L associates with *neCtnnb1* and *pCtnnb1* to regulate *Ctnnb1*’s transcription. **a** ChIP-seq tracks for enrichment of indicated *trans*-acting factors in *neCtnnb1* in developing Ncx. *neCtnnb1* is highlighted. **b** ChIP-seq tracks for ASH2L in indicated murine cells. *neCtnnb1* is highlighted. **c** ChIP-qPCR of E15.5 WT and *neCtnnb1*^KO^ Ncx using an anti-ASH2L antibody. *n* = 3 for WT Ncx and *n* = 3 for *neCtnnb1*^KO^ Ncx. Each point represents an individual brain. **d** RNA levels of *Ash2l* (left) and *Ctnnb1* (right) in WT and *neCtnnb1*^KO^ neocortical NPCs transfected with indicated vectors for two days. NPCs were derived from E12.5 Ncx. *n* = 3 for WT brains and *n* = 3 for *neCtnnb1*^KO^ brains. Each point represents an individual brain. **e** RNA levels of *Ash2l* (left) and *Ctnnb1* (right) in Neuro-2a cells transfected with indicated vectors for two days. Each point represents an independent experiment. **f** Schematic representation the location of *neCtnnb1* (pink shading) and conserved region (orange shading) marked by enrichment of H3K27ac, DNase I hypersensitivity and ASH2L in E14.5 brains. Data were obtained from ENCODE. Locations of sgRNA target sites and ChIP primer were indicated. **g**, **h** RNA levels of *Ctnnb1* in Neuro-2a cells transfected with indicated CRISPRa (**g**) and CRISPRi (**h**) vectors for two days. Each point represents an independent experiment. **i** Luciferase reporter assay in Neuro-2a cells transfected with indicated vectors for two days. Each point represents an independent experiment. Quantification data are shown as means ± SEM. Statistical significance was determined using two-way ANOVA followed by Sidak’s multiple comparisons test. (**c**, **d**, **e** and **i**); or using one-way ANOVA analysis (**g** and **h**). *P < 0.05, **P < 0.01, ***P < 0.001, and ****P < 0.0001. ns, not significant.

We next addressed which *trans*-acting factor(s) can associate with *neCtnnb1* to maintain *Ctnnb1*’s transcription. By analyzing ChIP-seq data of developing Ncx, several transcription factors and epigenetic modifiers were found to be enriched in *neCtnnb1* and/or *pCtnnb1*, including FOXP1, NEUROD2, NEUROG2, TBR2, SATB2, SMAD3, SOX2, ASH2L and EP300 (Fig. 5a). Literatures and ISH data deposited in the Allan Brain Atlas showed most of them are expressed in developing Ncx (Supplementary Fig. S5a). Among them, SOX2, SATB2, NEUROD2 and ASH2L were found to be enriched in both *neCtnnb1* and *pCtnnb1* and involved in Ncx neurogenesis. Particularly, a recent report found ASH2L, the COMPASS (complex of proteins associated with Set1) family histone methyltransferase co-factor, positively regulates *Ctnnb1*’s transcription and Ncx neurogenesis^44^. Sing-cell sequence data showed that, similar to *Tbr2*/*Eomes*, the transcript of *Ash2l* is highly enriched in IPs and immature PNs during mid-late neurogenesis (Supplementary Fig. S5b, c). In silico analyses showed ASH2L strongly associates with *neCtnnb1* and *pCtnnb1* in developing mouse Ncx but not in non-neural cells (Fig. 5b). Moreover, in *neCtnnb1*^KO^ cortical NPCs, the association of ASH2L with *pCtnnb1* was significantly compromised (Fig. 5c). Depletion of ASH2L could greatly downregulate *Ctnnb1*’s expression in Neuro-2a cells (Fig. 5e) or in WT Ncx NPCs (Fig. 5d), but not in *neCtnnb1*^KO^ NPCs (Fig. 5d). In comparison, knocking down *Sox2*, *Satb2*, or *NeuroD2* elicited no effect on *Ctnnb1* expression in Neuro-2a cells (Supplementary Fig. S5d–f), WT or *neCtnnb1*^KO^ NPCs (Supplementary Fig. S5h–j). However, knocking down *Yy1*, the gene encoding a transcription factor known to be a positive regulator of *Ctnnb1*^45^, could decrease *Ctnnb1* expression in Neuro-2a cells (Supplementary Fig. S5g), WT and *neCtnnb1*^KO^ NPCs (Supplementary Fig. S5k), demonstrating that YY1 maintains *Ctnnb1*’s transcription independent of *neCtnnb1*. Together, ASH2L associates with *pCtnnb1* and maintains *Ctnnb1*’s expression in an *neCtnnb1*-dependent manner.

We then investigated the precise and minimal region of *neCtnnb1* that sufficiently controls *Ctnnb1*’s transcription. Ten single-guide RNAs (sgRNAs) targeting the 5.19-kb-long region encompassing *neCtnnb1* (Fig. 5f) were individually used for CRISPRa and CRISPRi experiments in Neuro-2a cells. Interestingly, sgRNA#3 and #4, the two sgRNAs that target the conserved genomic region of *neCtnnb1* (*neCtnnb1*-CR), displayed the most prominent effects on inducing (Fig. 5g) or inhibiting (Fig. 5h) *Ctnnb1*’s transcription; and sgRNAs locating away from *neCtnnb1*-CR showed minor effects on regulating *Ctnnb1*’s expression. Multiple alignment of genomic sequences of the core conserved region in various species confirmed the high sequence homology across amniotes (Supplementary Fig. S6a, b). We thus cloned the 696-bp-long *neCtnnb1*-CR into the pGL3-pro vector. Compared to empty vector, pGL3-pro-CR displayed significantly higher luciferase activities in Neuro-2a cells. Moreover, overexpression ASH2L further enhanced the luciferase activities driven by *neCtnnb1*-CR (Fig. 5i).

**Fig. 6:**
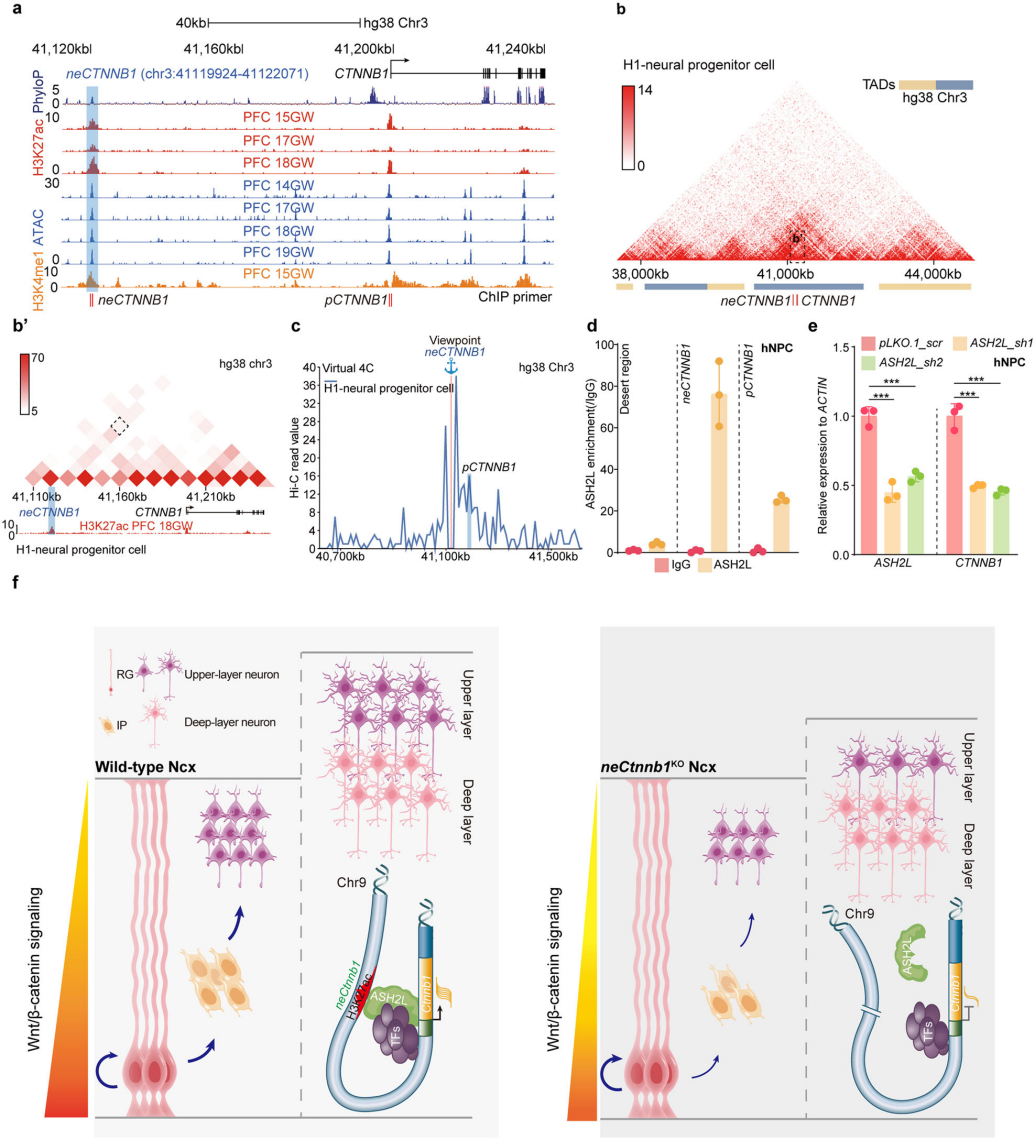
*neCTNNB1* is the human *CTNNB1* enhancer. **a** Schematic representation of human *CTNNB1* gene and the location of putative *neCTNNB1* enhancer (blue shading) marked by H3K27ac and H3K4me1 peaks and ATAC signals. **b** Hi-C data of H1-derived human neural progenitor cells (hNPC) were obtained from the 3D genome browser. Boundaries of TADs and locations of *neCTNNB1* and *CTNNB1* (red bars) are indicated below. **b**' Regions magnified from (**b**). *neCTNNB1* interacts with *CTNNB1* in H1-derived hNPC. **c** Virtual 4C of H1-derived hNPCs revealing interactions between *neCTNNB1* with *pCTNNB1*. **d** ChIP-qPCR of hNPC using an anti-ASH2L antibody. **e** RNA levels of *ASH2L* (left) and *CTNNB1* (right) in hNPC transfected with indicated virus for two days. Each point represents an independent experiment (**d**, **e**). **f** The working model. *neCtnnb1*, the upstream enhancer of *Ctnnb1*, controls production and transit-amplification of IPs, hence proper productions of UL PNs. ASH2L associates with *neCtnnb1* and positively regulates transcription of *Ctnnb1* in the *neCtnnb1*-dependent manner. In **e**, quantification data are shown as means ± SD. statistical significance was determined using two-way ANOVA followed by Sidak’s multiple comparisons test (**e**) *P < 0.05, **P < 0.01, and ***P < 0.001. ns not significant. PFC dorsal lateral prefrontal cortex.

In particular, *neCTNNB1*, the orthologous human genomic region of *neCtnnb1*, also bears essential enhancer features, such as chromatin accessibility determined by the transposase-accessible chromatin using sequencing (ATAC-seq), peaks for H3K27ac and H3K4me1, in developing human Ncx (Fig. 6a; Supplementary Fig. S7a). Hi-C data and virtual 4C analyses of developing human Ncx also indicated spatial proximity between *neCTNNB1* and *pCTNNB1* (Fig. 6b, c; Supplementary Fig. S7b, c). ChIP-qPCR experiments showed ASH2L strongly binds to *neCTNNB1* and *pCTNNB1* in human Ncx NPCs (Fig. 6d). Finally, depletion of ASH2L also significantly decreased expressions of *CTNNB1* (Fig. 6e).

## Disscussion

Although previous studies showed the canonical Wnt/β-catenin is involved in multiple aspects of neocortical development, its role in production and behaviors of IPs has not been fully understood. It was postulated that the Wnt/β-catenin signaling regulates both proliferation and differentiation of neural progenitors in vivo in a context-dependent manner, likely separated by either progenitor state and/or developmental time^16,46,47^. The canonical Wnt/β-catenin signaling displays a VZ-high to SVZ/IZ-medium to CP-low gradient in the developing Ncx, coinciding with the differentiation sequences from RGs to IPs, then to PNs^43^. Moreover, the strength of canonical Wnt/β-catenin signaling decreases over neurogenic time^48^, reflecting the progressive fate restriction of early to later RGCs and sequential generation of DL and UL PNs^46^.

Here we characterized an enhancer-driven canonical Wnt signaling predominantly active in Ncx SVZ, which maintains the generation and transit-amplification of IPs hence proper production of UL PNs (Fig. 6f). Boosting the canonical Wnt signaling in *neCtnnb1*-active cells greatly promotes IPs’ production and proliferation, leading to overproductions of UL PNs. Interestingly, this function exerted by *neCtnnb1* is independent of β-catenin’s roles in self-renewal of RGCs and the maintenance of the epithelial structure of the developing Ncx. Therefore, *neCtnnb1* spatiotemporally fine-tunes tones of Wnt signaling to control IP abundance and productions of UL PNs.

Most genetic studies supported the idea that the canonical Wnt/β-catenin signaling promotes RGC self-renewal while inhibits IP production and neuronal differentiation. Loss of β-catenin increases the Ncx IP population, and stabilization of β-catenin or expression of a dominant-active form of β-catenin promotes self-renewal of RGC and inhibits IP production^40,49^. But an in vitro study showed Wnt3a treatment increased the proportion of embryonic neocortex cells expressing TBR2 and decreased the percentage of RGCs^50^, which aligns with our findings and supports the notion that canonical Wnt/β-catenin signaling promotes IP production by RGCs. However, these and many other studies could not precisely manipulate the Wnt tone specifically in RGCs or IPs of the developing Ncx. For example, either depletion of RGCs or enhanced differentiation of IPs upon loss or gain of the Wnt/β-catenin signaling respectively could lead to the decrease of IP pool^16,51^. Even more complicated, manipulating the Wnt signaling by controlling expression levels or stability of β-catenin, overexpressing Wnt ligands, or bending the transactivating machinery using genetic or non-genetic means could result in distinct phenotypes, partly due to affected lateral expansion of RGCs and/or the disturbance of the Ncx epithelial structure^41,52,53^. We reason that although the overall Wnt/β-catenin signaling mediated by *neCtnnb1* is significantly compromised in developing Ncx upon loss of *neCtnnb1*, it reaches the threshold wherein the production and proliferation of IPs are mostly impacted but the self-renewal capacity and the epithelial architecture of RGCs remain uninfluenced.

As far as we know, *neCtnnb1* is the first enhancer that has been identified for *Ctnnb1*, the gene encoding β-catenin. *neCtnnb1* bears typical features for active enhancers during mid-late Ncx neurogenesis: *neCtnnb1* resides in the same chromatin subregion/compartment - TAD with the *Ctnnb1* gene and contacts with *pCtnnb1*, and is enriched with histone modifications including H3K27ac and H3K4me1. Moreover, the core-sequence of *neCtnnb1* is highly conserved across amniotes with Ncx-like structures in their brains (Supplementary Fig. S6b), coinciding with the presence of IPs in these animals^8,54,55^, which strongly points to *neCtnnb1*’s function in Ncx development and expansion. It remains to be elucidated whether the *neCtnnb1* sequences of higher animals, particularly the human *neCTNNB1*, display stronger activity than lower animals to boost IP production and division by enhancing the Wnt//β-catenin signaling.

An enhancer is a region of DNA that can be bound by *trans*-acting factors and associate with the promoter of its target genes to control the rate of transcription in particular cells at certain time points^56^. ASH2L has been found to be essential for Ncx development by sustaining expressions of multiple components of canonical Wnt signaling including *Ctnnb1*^44^. Interestingly, ablation of *Ash2l* in developing Ncx also caused depletion of IPs. Our study further revealed that ASH2L strongly associates with *neCtnnb1* and *pCtnnb1*. More importantly, we showed that the presence of *neCtnnb1* is required for ASH2L’s binding to *pCtnnb1*, hence for the proper expression of *Ctnnb1* in Ncx progenitors (Fig. 6f). In contrast, YY1, a reported transcriptional regulator of *Ctnnb1*^57^, positively controls *Ctnnb1*’s expression independent of the presence of *neCtnnb1* (Supplementary Fig. S5k). Future studies might unveil additional *trans*-acting regulators, such as epigenetic modifiers and transcription factors, which can cooperate with ASH2L to facilitate *Ctnnb1*’s transcription by modifying the *neCtnnb1*-*pCtnnb1* locus.

Previous high-throughput and reporter studies characterized numerous enhancers in the developing telencephalon^58^. Lately, the Encyclopedia of DNA Elements (ENCODE) project has identified more than 500,000 transposase-accessible regions during mouse fetal development with around five putative enhancers for one gene^59,60^. Nonetheless, only a fraction of these *cis*-elements with enhancer features have been functionally characterized^61^. Thus, there could be other enhancers of *Ctnnb1* to control its spatiotemporal expression in the context of development and/or pathophysiological scenarios. Enhancer mutations and aberrant activation are associated with developmental abnormalities such as polydactyly^62,63^, dysplasia of the sternum^27^, craniofacial malformation^64^, as well as malignant transformations^65^. SNPs inside core regions of *neCtnnb1* have yet to be associated with disorders related to neural development, but some might alter transcription factor binding (data not shown). The significance of these SNPs in physiological and pathological contexts deserves further investigation. Moreover, the activity and roles of *neCtnnb1* in adult brains, particular in lineages of adult neural stem cells, remain to be explored. Altogether, our findings advance the understanding of transcriptional regulation of *Ctnnb1*, and imply that canonical Wnt signaling controlled by *neCtnnb1* might be essential for Ncx expansion during evolution and development.

## Materials and methods

### Mice and genotyping

All mice studies were performed in accordance with protocols approved by the Animal Care and Ethical Committee at Wuhan University. All mice were in the C57BL/6 J background. The noon of the day when the vaginal plug was found was counted as embryo (E) day 0.5. *neCtnnb1*^KO^ mice were generated in the Beijing Biocytogen. *neCtnnb1-LacZ-iCre* mice were generated in the Shanghai Model Organisms Center Inc. BAT-Gal mice were kind gifts from Dr. Junlei Chang (Jackson Lab, stock number 005317). Mice with conditional activation of β-catenin were obtained by crossing *Ctnnb1*^*lox(ex3)*^ (a gift from Dr. Junlei Chang, MGI number: 1858008) with *neCtnnb1-LacZ-iCre*. Tamoxifen (TAM) was dissolved in corn oil as previously described^66^. To activate canonical Wnt/β-catenin signaling, TAM was injected into pregnant mice at indicated stages with a concentration of 100 mg/kg body weight. The primer set forward 5′-ccctgcccctgcatatagcatttga-3′/reverse 5′-ccccacctgtgatgctttgatgtct-3′/reverse 5′-atgctgtgtgagtgaccctactcct-3′ were used for *neCtnnb1*^KO^ mice genotyping. The primer set forward 5′-ggtagtggtccctgcccttgacac-3′/reverse 5′-acgtctggcaagttccgcgtcatcc-3′/reverse 5′-ctaagcttggctggacgtaaactc-3′ were used for *Ctnnb1*^*lox(ex3)*^ mice genotyping. The primer set forward 5′-atcctctgcatggtcaggtc-3′/reverse 5′-cgtggcctgattcattcc-3′ was used for BAT-Gal mice and *neCtnnb1-LacZ-iCre* mice genotyping. The band sizes for *neCtnnb1*^KO^ mice are 588 base pairs (bp) (wild-type allele), 477 bp (*neCtnnb1* knockout allele); ~1500 bp (wild-type allele), ~1300 bp (loxP allele) for *Ctnnb1*^*lox(ex3)*^ mice, and 315 bp (LacZ allele) for BAT-Gal mice and *neCtnnb1-LacZ-iCre* mice.

### Cell lines

HEK293T cells were gifts from Dr. Hongbing Shu (Wuhan University). Neuro-2a cells were purchased from the Cell Bank of Chinese Academy of Sciences, ReNcell CX human neural precursor cells (hNPCs) were purchased from EMD Millipore (Billerica, MA, USA, SCC008). HEK293T cells were maintained in indicated culture media (DMEM, Gbico) containing 10% fetal bovine serum (FBS) (Life Technologies), Neuro-2a cells were maintained in indicated culture media (MEM, Gbico) containing 10% FBS, non-essential amino acid solution (1×, Gbico, 10370021) and 1 mM sodium pyruvate (Gbico, 11360070). ReNcell CX hNPCs were plated onto laminin (Sigma-Aldrich, 11243217001) coated cell culture plate and maintained in culture media (DMEM/F12, Gbico) containing N2 and B27 supplements (1×, Life Technologies), human recombinant FGF2, and EGF (20 ng/mL each; Life Technologies) in a CO_2_ cell culture incubator^67^. Mouse neocortical neural progenitor cells (NPCs) were enriched from E12.5 mouse cortex, cultured on ultra-low-attachment plates (Corning, New York, United States) and maintained in indicated culture media (DMEM/F12, Life Technologies) containing N2 and B27 supplements (1×, Life Technologies), 1 mM Na-pyruvate, 1 mM N-acetyl-L-cysteine (NAC), human recombinant FGF2, and EGF (20 ng/mL each; Life Technologies).

### Tissue fixation and sectioning

Pentobarbital sodium [0.7% (w/v), 105 mg/kg of body weight] in 0.9% sodium chloride was peritoneally injected into pregnant mice carefully. Embryos were sequentially removed from uteri, and tails removed for genotyping. Brains were dissected out from embryos in cold phosphate-buffered saline (PBS). For immunofluorescent staining and in situ hybridization, brains were immersed in 4% paraformaldehyde (PFA) overnight at 4 °C; for X-Gal staining, brains were immersed in 4% PFA for 10 min (brains) or 30 min (whole body) at room temperature. For adult mice, mice were deeply anesthetized with intraperitoneal injection of 0.7% (w/v) pentobarbital sodium solution followed by perfusion with 4% PFA. Brains were dissected out and immersed in 4% PFA overnight at 4 °C. The next day, 4% PFA was replaced with 20% (w/v) sucrose for embryo brains or 30% (w/v) sucrose for adult brains overnight at 4 °C. For sectioning, brains were embedded in OCT (SAKURA) and cut at 20 μm for adult brains and X-Gal staining brains, and 14 μm for other stages with a cryotome (Leica CM1950) onto coated slides.

### Chromosome conformation capture (3C)

The 3C assay was conducted essentially as described previously^68,69^. 1 × 10^7^ Neuro-2a cells were cross-linked with 2% formaldehyde for 10 min at room temperature. Next, reaction tubes were transferred into ice and added 0.125 M glycine for 5 min to quench the cross-linking reaction. The pellet was lysed in ice-cold 3C lysis buffer (10 mM Tris-HCl, pH 7.5; 0.1 mM EGTA; 10 mM NaCl; 5 mM MgCl_2_; 1× protease inhibitor) and incubated on ice for 10 min. Nuclei were pelleted by centrifugation at 400× *g* for 5 min at 4 °C. Pelleted nuclei were taken up to a new tube with 500 μL 1.2× restriction enzyme buffer (NEB) and 7.5 μL 20% (w/v) SDS on rocker for 1 h at 37 °C. 1 h later, 50 μL of 20% (v/v) Triton X-100 was added to tube followed by 1 h incubation at 37 °C with rotation. Pelleted nuclei were digested by *Bam*H I (NEB) overnight at 37 °C with rotation. The next day, 40 μL of 20% (w/v) SDS were added to tube for 25 min at 65 °C with rotation. Digested nuclei were transfer to a new tube followed by adding 6.125 mL of 1.15× ligation buffer (660 mM Tris-HCl, pH 7.5; 50 mM DTT; 10 mM ATP, 50 mM MgCl_2_) and 375 μL 20% (v/v) Triton X-100 for 1 h at 37 °C with rotation. 700 U T4 DNA ligase (Takara Bio) was added to the tube and incubate for 4 h at 16 °C followed by 30 min at room temperature. Next, 300 μg proteinase K was added to reverse cross-linked chromatin at 65 °C for overnight. The next day, 300 μg RNase A (TARAKA) was added and incubated at 37 °C for 40 min. The fragments of DNA were extracted by phenol-chloroform extraction and analyzed by RT-qPCR. The specificity and efficiency of all 3C primers can be verified by digestion and ligation of BAC DNA containing the target region. The frequency of cross-linking was calculated using the parameters of BAC DNA standard curve. DNA: value = 10^(Ct − b/a)^ (b: intercept; a: slope). Finally, these values were normalized to *Gapdh* to generate the relative cross-linking frequency.

### CRISPR/dCas9-mediated transcription activation (CRISPRa) and interference (CRISPRi) assay

Clustered regularly interspaced short palindromic repeat (CRISPR) interference (CRISPRi), in which a nuclease-null Cas9 (dCas9) is fused to the Krüppel-associated box (KRAB) repressor, can specifically silence target endogenous gene expression. CRISPR activation (CRISPRa), in which a nuclease-null Cas9 (dCas9) is fused to transcriptional activators like VP64 domain, enables efficient increase in target endogenous gene expression. CRISPRi and CRISPRa assays were performed according to published procedures^70,71^. SgRNAs were designed to target *neCtnnb1* by using online tool (https://zlab.bio/guide-design-resources). SgRNAs were cloned into sgRNA (MS2) cloning vector (Addgene, #61424). For CRISPRi assays, vectors of sgRNA and pHR-SFFV-dCas9-BFP-KRAB (Addgene, #46911) were transfected into cells. For CRISPRa assays, vectors of sgRNA, MS2-P65-HSF1_GFP (Addgene, #61423) and dCAS9-VP64_GFP (Addgene, #61422) were transfected into cells. Fourty-eight hours after transfection, cells were collected to extract RNAs and the expression of *Ctnnb1* was quantified by RT-qPCR.

### RNA isolation, cDNA synthesis and quantitative RT–PCR (qPCR)

Total RNAs were prepared using the RNAiso Plus (TAKARA) according to the manufacturer’s protocols. Tissues or cells were lysed with 1 mL or 500 μL RNAiso Plus in DNase/RNase-free EP tubes on ice, followed by adding 200 μL or 100 μL chloroform to achieve phase separation. After shaking vigorously, tubes were centrifuged at 12,000× rpm for 15 min at 4 °C, and then transfer aqueous phase to new tubes. The aqueous phase was mixed with equal volumes of isopropyl alcohol to obtain RNA. Precipitation was resuspended with an appropriate DNase/RNase-free water. Complementary DNAs (cDNAs) were synthesized by HiScript® II Q RT SuperMix for qPCR kit (Vazyme; R222-01). qPCR primers were designed by PrimerBank^72^. cDNAs were used to detect different genes with 2× SYBR Green qPCR master mix (Bimake). Amplifications were performed using the CFX Connect Real-Time PCR Detection System (Bio-Rad) with a final volume of 10 μL under the following condition: 5 min at 95 °C and then 40 cycles at 95 °C for 15 s and 60 °C for 20 s. Relative expression levels for target genes were calculated using the 2^−ΔΔCt^ method^73^, normalized to the *Gapdh* or *ACTIN* housekeeping gene.

### X-Gal staining

For frozen sections, sections were fixed in fresh cold fixative (0.2% PFA) in buffer L0 (0.1 M PIPES buffer (pH 6.9), 2 mM MgCl_2_, 5 mM EGTA) for 10 min. Rinse the slides in PBS plus 2 mM MgCl_2_ on ice, followed by a 10 min wash in the same solution. Place slides in detergent rinse [0.1 M PBS (pH 7.3), 2 mM MgCl_2_, 0.01% sodium-deoxycholate, 0.02% Nonidet P-40] on ice for 10 min. Slides were then moved to a freshly made and filtered X-Gal staining solution [0.1 M PBS (pH 7.3), 2 mM MgCl_2_, 0.01% sodium-deoxycholate, 0.02% Nonidet P-40, 5 mM K_3_Fe(CN)_6_, 5 mM K_4_Fe(CN)_6_·3H_2_O and 1 mg/ml X-Gal]. Sections were incubated at 37 °C from a few minutes to overnight in the dark. Sections were rinsed with water to stop the reaction. Sections were dehydrated with gradient ethanol and xylene sequentially, and mounted with neutral balsam. For whole body or brains, tissues were immersed in 4% PFA 10 min (brains) or 30 min (whole body) at room temperature. Rinse fixed tissues in detergent rinse three times at room temperature for 15–30 min. Tissues were then transferred to freshly made and filtered X-Gal staining solution. The staining time depends on the size of the sample and the strength of bacterial β-Gal expression. After staining, embryos or brains were washed three times in PBS and photographed^74^.

### Nissl staining

Brain sections were stained in 0.25% cresyl violet solution (Sigma-Aldrich) for 15 min at 65 °C. Then, sections were decolorized in ethanol, the decolorization time depends on the strength of color. After decolorizing, sections were dehydrated in ethanol for 5 min, followed by a 10 min wash in xylene and mounted in a neutral balsam.

### Immunohistochemical staining

Frozen brain sections were heated to 95°C in citrate antigen retrieval solution (pH 6.0) for 25 min to deactivate endogenous peroxidase. When the temperature of the solution drops to room temperature, sections were rinsed three times in PBS and blocked in solution 1 [PBS with 0.5% Triton X-100 and 5% bovine serum albumin (BSA)] for 1 h at room temperature. Sections were then incubated overnight at 4 °C with primary antibodies in solution 1. The next day, sections were incubated with appropriate secondary antibodies for 1 h at room temperature. Then, sections were incubated with the avidin-biotin-peroxidase complex (A:B = 1:50; VECTASTAIN Elite ABC system, Vector Labs). Peroxidase was reacted in solution 2 (tris-HCl (pH 7.2) with 5 mg/ml 3,3'-diaminobenzidine and 0.075% H_2_O_2_). The sections were dehydrated, washed in xylene and mounted in a neutral balsam.

### Immunofluorescence

Frozen brain sections were dried at temperature and heated to 95 °C in citrate antigen retrieval solution (pH 6.0) for 25 min. For BrdU staining, sections were incubated with 2 N HCl 30 min at room temperature. Sections were blocked in blocking buffer (3% heat-inactivated normal goat serum, 0.1% Triton X-100 and 0.1% BSA in 10 mM Tris-HCl, pH 7.4; 100 mM NaCl) for 1 h at room temperature. Sections were then incubated overnight at 4 °C with primary antibodies [rat anti-BrdU (1:500; Abcam, ab6326), rabbit anti-TBR2 (1:1000; Abcam, ab23345), rabbit anti NEUROD2 (1:500; Abcam, ab104430), chicken anti- beta Galactosidase (1:500; Immunology Consultants Laboratory, CGAL-45A-Z) rat anti-TBR2 (1:1000; Thermo Fisher Scientific, 14-4875-82), mouse anti-FOXP2 (1:250; Sigma-Aldrich, AMAB91361), rat anti-CTIP2 (1:500; Abcam, ab18465), mouse anti-SATB2 (1:500; Abcam, ab51502), rabbit anti-CUX1 (1:100; Santa Cruz Bio- technology, sc-13024) and rabbit anti-PAX6 (1:500; Millipore, AB2237)] in blocking buffer. The next day, sections were incubated with appropriate secondary antibodies (Alexa Fluor 488-conjugated anti-mouse, Alexa Fluor 488-conjugated anti-rat, Alexa Fluor 488-conjugated anti-rabbit, Alexa Fluor 555-conjugated anti-mouse, Alexa Fluor 555-conjugated anti-rabbit and Alexa Fluor 647-conjugated anti-rabbit; Thermo Fisher Scientific; 1:1000) for 1 h at room temperature. Sections were mounted with anti-fade (1×; Thermo Fisher Scientific) solution with 4′,6-diamidino-2-phenylindole (DAPI, 0.1 μg/ml) in PBS. All comparative expression levels of immunofluorescence were obtained at the same collective conditions.

### 5-Ethynyl-2′-Deoxyuridine (EdU) staining

Proliferation of cells was investigated with BeyoClick^TM^ EdU Cell Proliferation Kit (C0075S, Beyotime, China) according to the manufacturer’s protocols. In brief, frozen brain sections were dried at temperature and permeated with 0.3% Triton X-100 in PBS for 30 min. After that, the sections were incubated with EdU working solution for 1 h at 37 °C in the dark. After incubation, regular immunofluorescence staining can be followed.

### In situ hybridization (ISH)

Templates for antisense RNA probes were cloned from mouse cDNA with specific primers for *Ctnnb1*. Then, templates were cloned into the pGEM-T Easy vector (Promega). Vector with target template was linearized by appropriate restriction enzyme and then transcribed to DIG-labeled probes by using the DIG-RNA Labeling Mix (Roche). Cryosections were dried in a hybridization oven for 15 min at 50 °C, followed by fixing with 4% PFA for 20 min at room temperature. Then, sections were permeabilized with 2 μg/mL proteinase K (Sigma) in PBS for 10 min at room temperature and acetylation in 0.1 M TEA (triethanolamine) for 10 min at room temperature. After permeabilization and acetylation, sections were blocked in hybridization buffer (5× SSC, 5× Denharts; 500 μg/mL herring sperm DNA; and 250 μg/mL yeast RNA; 50% deionized formamide) for 3 h at room temperature followed by incubating with a DIG-labeled probe diluted (0.2 ng/μL) in hybridization buffer overnight at 65 °C in a hybridization oven. Sections were washed with 0.1× SSC for four times (20 min each) in a hybridization oven at 65 °C, followed by treating with ribonuclease A (TAKARA) (20 μg/mL) for 20 min at 37 °C and then blocked with 10% normal sheep serum in Buffer B1 (0.1 M Tris-HCl, pH 7.4; 150 mM NaCl) for 3.5 h at room temperature. Sections were incubated with 1:5000 dilution of anti-DIG antibody (Roche) overnight at 4 °C. The next day, sections were washed with Buffer B3 (0.1 M Tris-HCl; 0.1 M NaCl; 50 mM MgCl_2_; 0.1% Tween-20, pH 9.5) for three times (10 min each) at room temperature, followed by colorization with BCIP/NBT (bromochloroindolyl phosphate/nitro blue tetrazolium) (Roche) containing B3 solutions at room temperature in dark. The colorization time depends on the strength of target genes expression. Sections were dehydrated with gradient ethanol and xylene sequentially, and mounted with neutral balsam. The probe of *Ctnnb1* were forward 5′-cacgactagttcagctgcttgt-3′/reverse 5′-tccacacatgaacatctccttc-3 according to the ALLEN BRAIN MAP (https://portal.brain-map.org/).

### In utero electroporation (IUE) of developing neocortices

Pregnant WT and *neCtnnb1*^KO^ mice with E14.5 embryos were anesthetized by peritoneally injection of pentobarbital sodium (70 mg/kg), and the uteri were exposed through a 2 cm midline abdominal incision. Embryos were carefully pulled out using ring forceps through the incision and placed on sterile gauze wet with 0.9% sodium chloride. Plasmid of pCIG (1 μg/μL, prepared using Endo Free plasmid purification kit, Tiangen) mixed with 0.05% Fast Green (Sigma) was injected through the uterine wall into the telencephalic vesicle. Five electric pulses (36 V, 50 ms duration at 1 s intervals) were generated using CUY21VIVO-SQ (BEX) and delivered across the head of embryos using 5 mm forceps-like electrodes (BEX). The uteri were then carefully put back into the abdominal cavity, and both peritoneum and abdominal skin were sewed with surgical sutures. The whole procedure was completed within 30 min. Mice were warmed on a heating pad until they regained consciousness and were treated with analgesia (ibuprofen in drinking water) until sacrifice at E18.5.

### ChIP-qPCR assay

ChIP-qPCR was conducted essentially as described previously^75^. 100 mg forebrain cortex (gently homogenized to cell dispersion in ice-cold PBS with protease inhibitors) or 1 × 10^7^ cells were cross-linked with 1% formaldehyde for 10 min at room temperature and quenched by adding 0.125 M glycine for 5 min. Cells were then washed with ice-cold PBS twice (10 min each). Next, cells were harvested in 500 μL lysis buffer (50 mM Tris-HCl, pH 8.0, 0.5% SDS, 5 mM EDTA) and 500 μL digestion buffer (50 mM Tris-HCl, pH 7.6; 1 mM CaCl_2_, 0.2% Triton X-100) plus 1400 U micrococcal nuclease (NEB; M0247S) for 20 min at 37 °C, followed by adding 5 μL 0.5 M EDTA to stop reaction and incubating on ice for 5 min. Sonicate cells in EP tubes with power output 100 W, 3 min (cells) or 7 min (cortex), 0.5 s on, 0.5 s off on ice. The agarose gel ensures that the size of DNA fragments was proper. One percent of the sonicated lysate was taken as the input. The rest of the sonicated lysates was diluted into 0.1% SDS using dilution buffer (20 mM Tris-HCl, pH 8.0; 150 mM NaCl; 2 mM EDTA; 1% Triton X-100) and divided into two parts for IgG and target protein immunoprecipitation. Samples were incubated with 25 μL pre-washed protein G agarose beads and 2 μg anti-ASH2L (Bethyl Laboratories; A300-489A) or 25 μL anti-Flag affinity gel (Bimake; B23101) overnight at 4 °C on rocker. The next day, beads were wash with Wash Buffer I (20 mM Tris-HCl, pH 8.0; 1% Triton X-100; 2 mM EDTA; 150 mM NaCl; 0.1% SDS), Wash Buffer II (20 mM Tris-HCl, pH 8.0; 1% Triton X-100; 2 mM EDTA; 500 mM NaCl; 0.1% SDS), Wash Buffer III (10 mM Tris-HCl, pH 8.0; 1 mM EDTA; 0.25 M LiCl; 1% NP-40; 1% deoxycholate) and TE buffer. Beads were resuspended in elution buffer (0.1 M NaHCO_3_, 1% SDS, 20 µg/mL proteinase K) to elute the DNA. The elution was incubated at 65 °C for overnight to reverse cross-linked chromatin. DNA fragments were extracted with DNA purification kit (TIANGEN). The purified DNA was quantified by RT-qPCR.

### 3D genome browser analysis

The Hi-C and virtual 4C data from cells or tissues were obtained from the 3D Genome Browser^76^ (http://3dgenome.fsm.northwestern.edu/). In addition, TADs were identified to screen for potentially interacting genes in cells or tissues.

### Luciferase reporter assay

The luciferase reporter assay was performed as described^77^. Briefly, the 696-bp conserved region of *neCtnnb1* (chr9:120,869,524-120,870,219, CR) was cloned from mouse brain genomic DNA and subcloned into pGL3-promoter vector (Promega). Neuro-2a cells were transfected with pGL3-promoter vector and pGL3-pro-CR vector or pCDH-ASH2L along with pGL3-promoter vector and pGL3-pro-CR vector. *Renilla* basic vector was cotransfected as a control for normalization of luciferase activity. Luciferase activity was measured 48 h after transfection using a Promega Dual-Glo assay kit, as per the manufacturer’s instructions.

### Quantification and statistical analysis

Sections used for quantification were position-matched for wild-type and experimental brains. Images were binned against dorsal-ventral position to quantify the intensity of layer-specific distributed ISH or LacZ signals of cortex. The signal intensity of those regions was generated using ImageJ as described previously^78,79^. Statistical tests were performed using GraphPad Prism (version 8.0.2). Data are presented as means ± SEM or ± SD. Unpaired two-tailed Student’s *t-*test were used for analysis between two groups of equal variances by *F*-tests. When the *F*-test of equal variance failed, the Welch *t*-tests were used. Data comparison of three or more groups with control groups were analyzed using one-way ANOVA followed by Dunnett’s multiple comparison test. Data comparison of three or more groups among each other were analyzed using one-way ANOVA followed by Tukey’s multiple comparison test. Statistically significant was considered by *P* ≤ 0.05 (*P < 0.05, **P < 0.01, and ***P < 0.001).

## Supplementary information

Supplementary information

## Data Availability

All data are available in the paper and/or the supplementary materials.
