# Supplementary material for: A Ctnnb1 enhancer regulates neocortical neurogenesis by controlling the abundance of intermediate progenitors
Source: Cell Discov. 2022 Aug 2;8:74. doi: 10.1038/s41421-022-00421-2 (PMC9343459; doi:10.1038/s41421-022-00421-2)
Supplement: Supplementary file 1 — Supplementary information [file 41421_2022_421_MOESM1_ESM.pdf]

Supplementary information

A *Ctnnb1* enhancer regulates neocortical neurogenesis by controlling the abundance of intermediate progenitors

Junbao Wang[1,#], Andi Wang[1,#], Kuan Tian[1], Xiaojiao Hua[1], Bo Zhang[1], Yue Zheng[1], Xiangfei Kong[1], Wei Li[1], Lichao Xu[1], Juan Wang[2], Zhiqiang Li[1], Ying Liu[1,*], Yan Zhou[1,*]

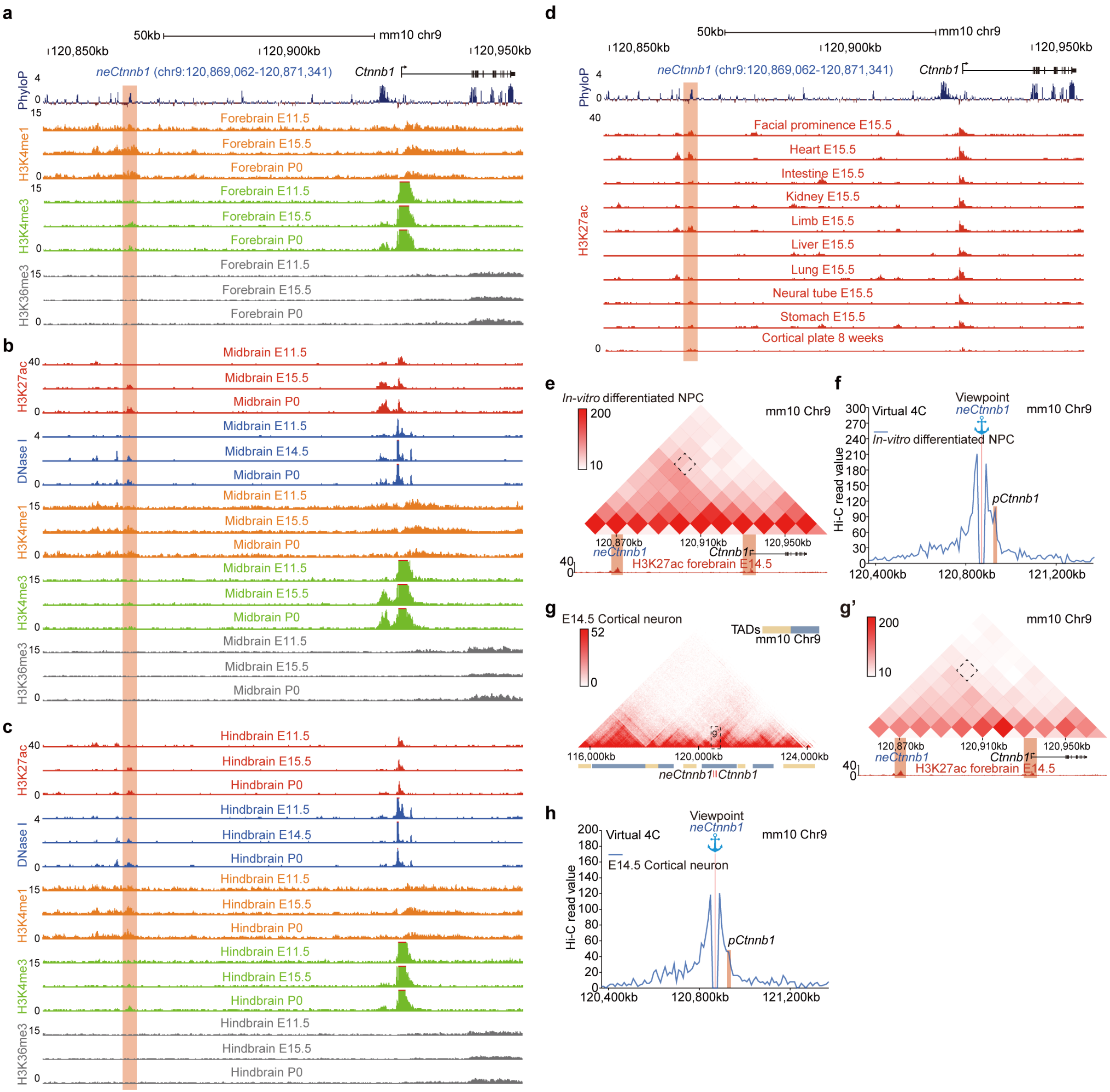

Supplementary Figure S1. *neCtnnb1* is a putative enhancer upstream of *Ctnnb1*.

a-c Schematic representation of the upstream region of mouse *Ctnnb1* gene and the location of putative enhancer *neCtnnb1* (orange shading). Enrichment of indicated signals in developing forebrains (**a**), midbrains (**b**) and hindbrains (**c**) were shown. Data were obtained from ENCODE. **d** Enrichment of H3K27ac ChIP-seq signals in indicated tissues of E15.5 embryos. Data were obtained from ENCODE. **e-f** The Hi-C data (**e**) and virtual 4C (**f**) of *in-vitro* differentiated NPCs revealing the interaction between *neCtnnb1* and *pCtnnb1*. **g** The Hi-C data of E14.5 cortical neurons were obtained from the 3D genome browser. Boundaries of TADs and locations of *neCtnnb1* and *Ctnnb1* gene (red bars) are indicated below. **g'** Regions magnified from (**g**). Boxed area indicating the interaction between *neCtnnb1* and *pCtnnb1*. **h** Virtual 4C of E14.5 cortical neurons revealing the interaction between *neCtnnb1* and *pCtnnb1*.

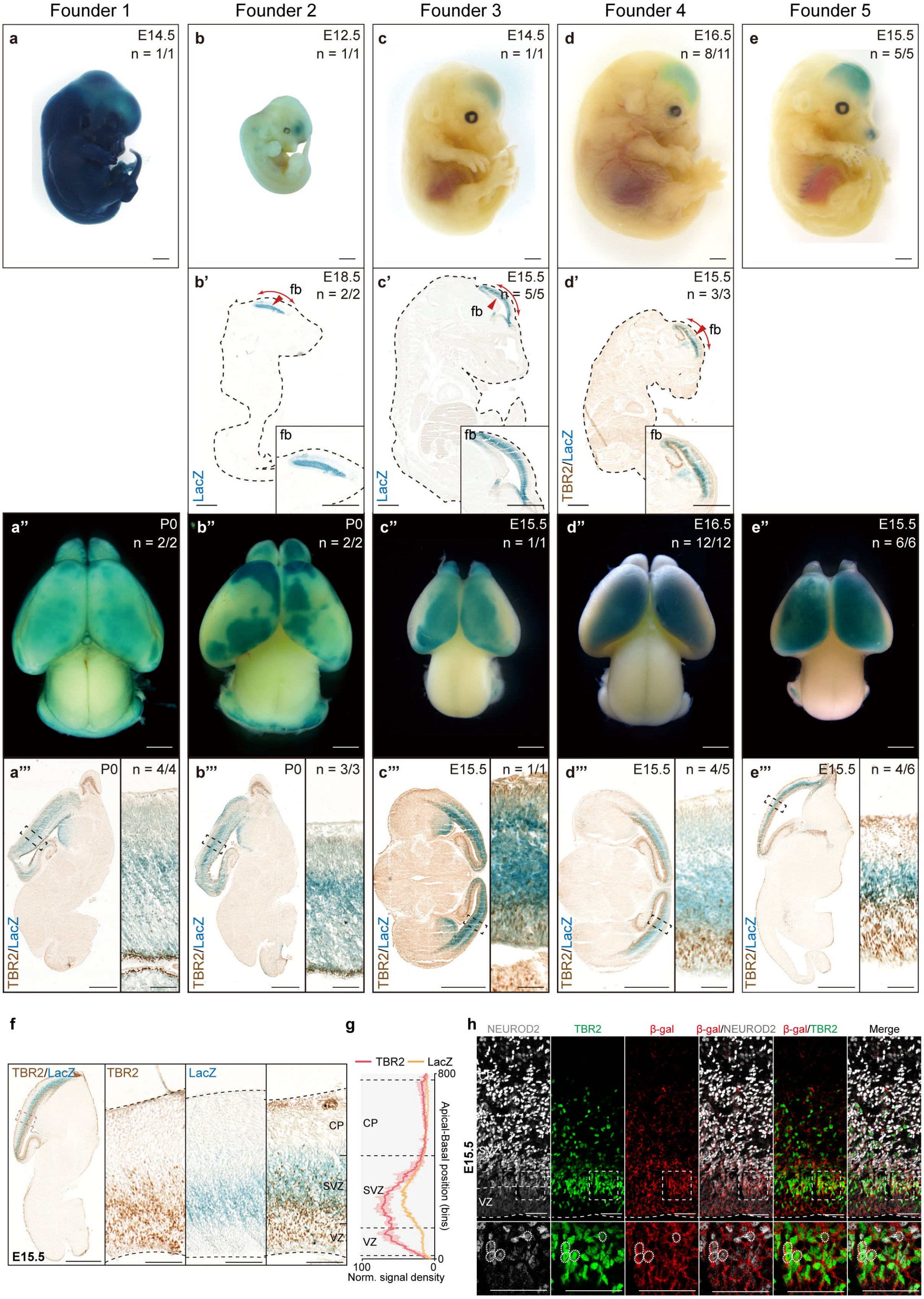

Supplementary Figure S2. *neCtnnb1* is predominantly active in developing neocortex.

a-e X-Gal staining (blue) of whole embryos (**a**-**e**), sagittal whole-body sections (**b’-d’**), brains (**a”-e”**) and sagittal/coronal brain sections (**a’”-e’”**) of *neCtnnb1-LacZ-iCre* reporter mice. Due to the influence of integration sites and copy numbers on expression, the reporter activity in a specific tissue was only considered reliable when it was found in at least two or more independent individual embryos. **f** E15.5 sagittal brains sections of *neCtnnb1-LacZ-iCre* mice were sequentially stained with LacZ/β-Gal (blue) for *neCtnnb1* activity and TBR2 (brown), with boxed Ncx regions magnified on right panels. **g** Quantifications of normalized signal density of β-Gal and TBR2 signals in (**f**). **h** Immunofluorescence of NEUROD2 (gray), TBR2 (green), and β-Gal (red) on coronal sections of E15.5 *neCtnnb1-LacZ-iCre* Ncx. Scale bars, 1mm (**a**-**e**, **b**’-**d**’, **a**”-**e**”, **a**’”-**e**’” and **f**), 100 μm (magnified views in **a**’”-**e**’” and **f**), 50 μm (**h**).

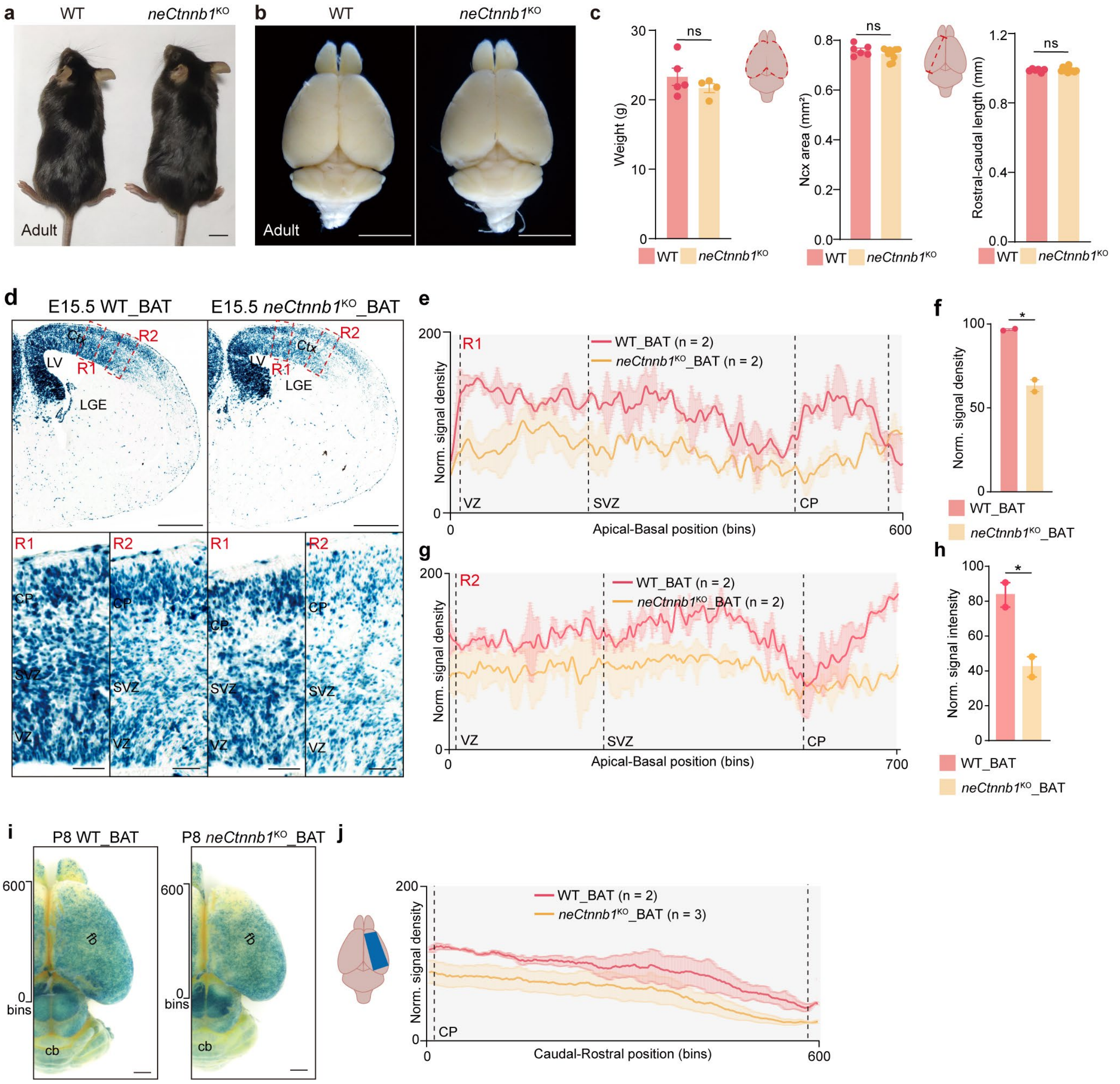

Supplementary Figure S3. Knockout of *neCtnnb1* compromised *Ctnnb1's* reporter activity in developing neocortices.

a Representative images of adult (8 weeks) WT and *neCtnnb1*^{KO} mice. **b** Dorsal views of adult WT and *neCtnnb1*^{KO} mouse brains. **c** Quantifications of adult WT and *neCtnnb1*^{KO} mice in body weight, neocortex area and length. n = 5 for WT brains and n = 4 for *neCtnnb1*^{KO} brains. Each point represents an individual brain. **d** X-Gal staining (blue) on E15.5 coronal sections of WT_BAT (left) and *neCtnnb1*^{KO}_BAT (right) Ncx, with boxed regions (R1s and R2s) magnified on the bottom. **e**-**h** Quantification of normalized signal density of LacZ in two boxed regions of (**d**). n = 2 for WT_BAT brains and n = 2 for *neCtnnb1*^{KO}_BAT brains. Each point represents an individual brain. **i** X-Gal staining (blue) in P8 WT_BAT (left) and *neCtnnb1*^{KO}_BAT (right) whole mount brains. **j** Quantification of normalized signal density of LacZ in boxed regions of (**i**). n

= 2 for WT_BAT brains and n = 3 for *neCtnnb1*^{KO}_BAT brains. Each point represents an individual brain. Quantification data are shown as mean ± SEM. Statistical significance was determined using an unpaired two-tailed Student's *t*-test (**c**, **f** and **h**) $*P < 0.05$, $**P < 0.01$, and $***P < 0.001$. ns, not significant. Scale bars, 1 cm (**a** and **i**), 500 μm (**b** and **d**), 50 μm (magnified views in **d**).

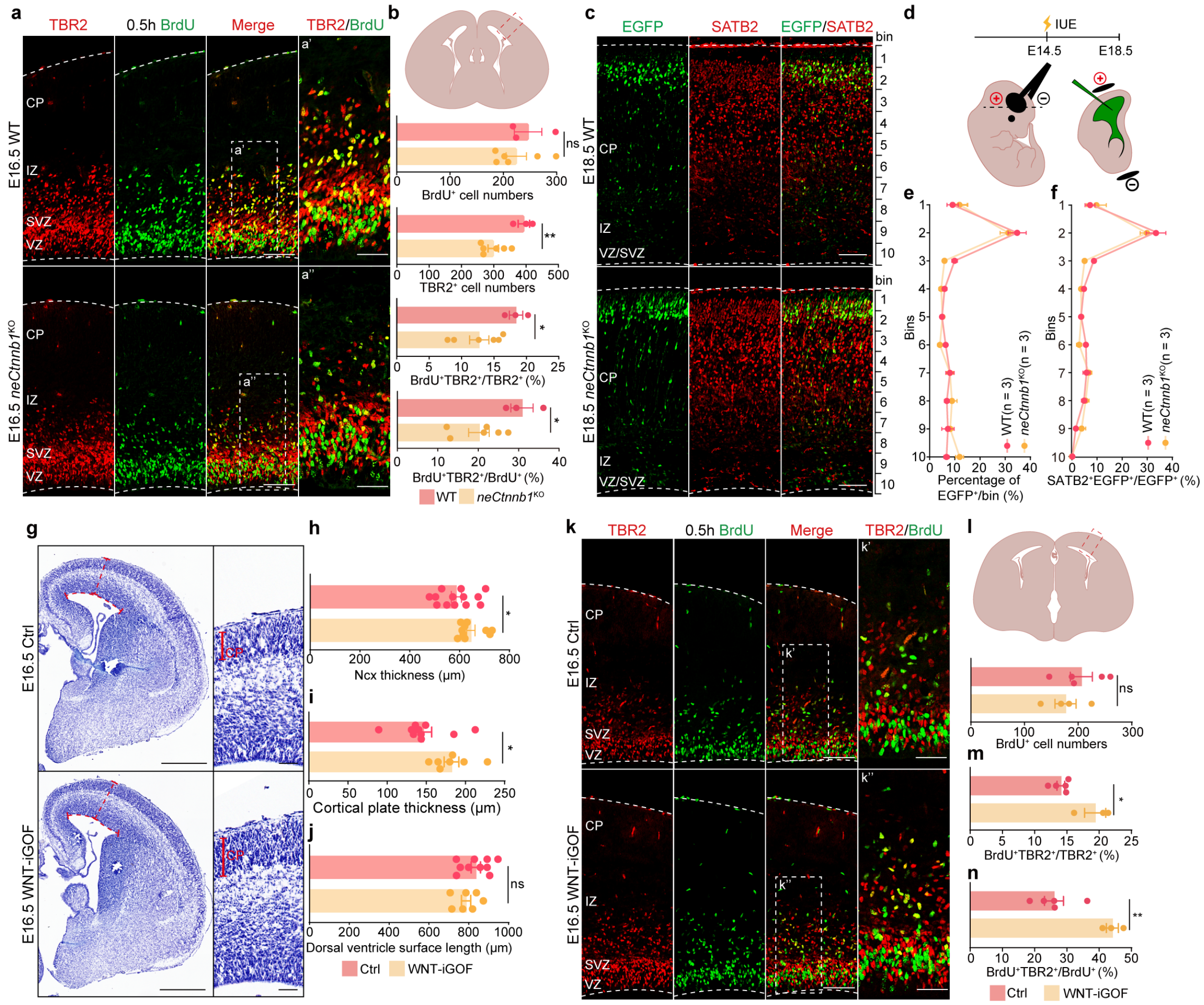

Supplementary Figure S4. *neCtnnb1* regulates neocortical neurogenesis and IP divisions.

a-b Immunofluorescence (**a**) and quantification (**b**, top) of BrdU+ (green) and TBR2+ (red) cells on boxed area of coronal sections (**b**) of E16.5 WT and *neCtnnb1*^{KO} Ncx. Pregnant mice were injected with BrdU 30 min before sacrifice. n = 3 for WT brains and n = 6 for *neCtnnb1*^{KO} brains. Each point represents an individual brain. **c** Immunofluorescence of SATB2+ (red) and EGFP+ (green) cells on E18.5 coronal sections of WT and *neCtnnb1*^{KO} Ncx. Neocortex were electroporated with EGPF-expressing constructs at E14.5. **d** Schematic diagram illustrating the strategy of *in utero* electroporation (IUE) of WT and *neCtnnb1*^{KO} embryos. **e-f** Neocortices shown in (**c**) were divided into 10 bins of equal height and fixed width, and EGFP+ (green) cells in each bin were counted and quantified. n = 3 for WT brains and n = 3 for *neCtnnb1*^{KO} brains. Each point represents an individual brain. **g** Nissl-staining on E16.5 coronal sections of Ctrl and WNT-iGOF Ncx. **h** Comparison of neocortical thickness of E16.5 Ctrl and WNT-iGOF mice. n = 13 for Ctrl brains and n = 10 for WNT-iGOF brains. Each point represents an individual brain. **i** Comparison of cortical plate thickness of E16.5

Ctrl and WNT-iGOF mice. n = 10 for Ctrl brains and n = 7 for WNT-iGOF brains. Each point represents an individual brain. **j** Comparison of dorsal ventricle surface length of E16.5 Ctrl and WNT-iGOF mice. n = 10 for Ctrl brains and n = 7 for WNT-iGOF brains. Each point represents an individual brain. **k-n** Immunofluorescence (**k**) and quantification (**l-n**) of TBR2+ (red) and BrdU+ (green) cells on boxed area of coronal sections (**l**) of E16.5 Ctrl and WNT-iGOF Ncx. Pregnant mice were injected with BrdU 30 min before sacrifice. Each point represents an individual brain. Quantification data are shown as mean ± SEM. Statistical significance was determined using unpaired two-tailed Student's *t*-test (**b**, **h**, **i**, **j**, **l**, **m** and **n**); two-way ANOVA followed by Sidak's multiple comparisons test (**e** and **f**). *$P < 0.05$, **$P < 0.01$, ***$P < 0.001$, and ****$P < 0.0001$. ns, not significant. Scale bars, 500 μm (**g**), 100 μm (**a**, **c**, **k** and magnified views in **g**), 50 μm (magnified views in **a** and **k**).

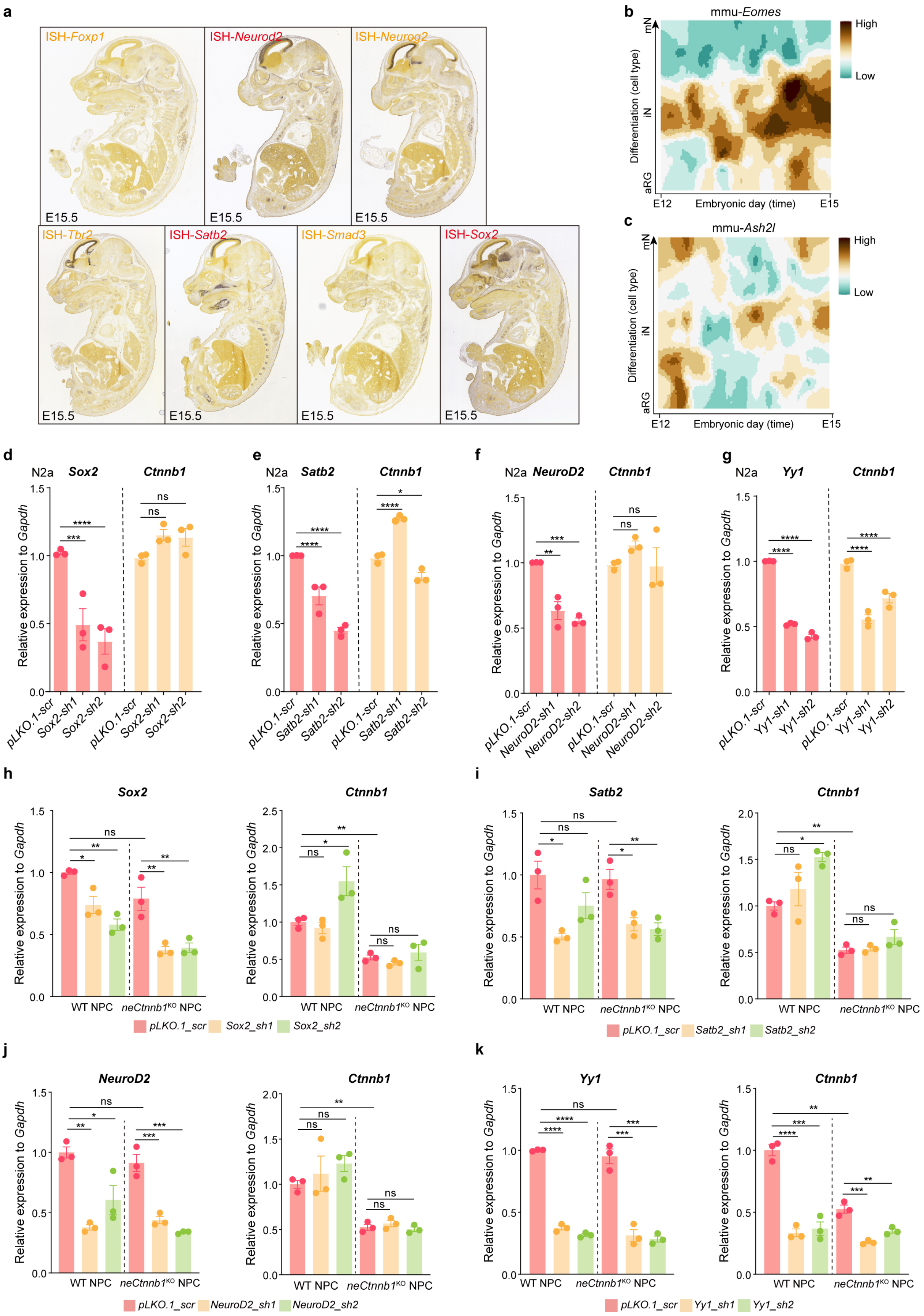

Supplementary Figure S5. Effects of selected *trans*-acting factors on

expressions of *Ctnnb1*.

a Representative images showing *in situ* hybridization (ISH) of *Foxp1, Neurod2, Neurog2, Tbr2, Satb2, Smad3* and *Sox2* on sagittal sections of E15.5 mouse embryos. Data were extracted from the Allan Brain Atlas. **b-c** Expression patterns of *Eomes* (**b**) and *Ash2l* (**c**) during mouse neocortical neurogenesis. Visualized single-cell RNA-seq data were extracted from Humous.org (http://www.humous.org/). **d-g** RNA levels of *Sox2* (**d**), *Satb2* (**e**), *NeuroD2* (**f**), *Yy1* (**g**) and *Ctnnb1* in Neuro-2a transfected with indicated vectors for two days. Each point represents an independent experiment. **h-k** RNA levels of *Sox2* (**h**), *Satb2* (**i**), *NeuroD2* (**j**), *Yy1* (**k**) and *Ctnnb1* in WT and *neCtnnb1*^{KO} neocortical NPCs transfected with indicated vectors for two days. NPCs were derived from E12.5 Ncx. n = 3 for WT brains and n = 3 for *neCtnnb1*^{KO} brains. Each point represents NPCs derived from an individual brain. Quantification data are shown as mean ± SEM. Statistical significance was determined using two-way ANOVA followed by Sidak's multiple comparisons test. (**d**-**k**). $^{*}P < 0.05$, $^{**}P < 0.01$, $^{***}P < 0.001$, and $^{****}P < 0.0001$. ns, not significant.

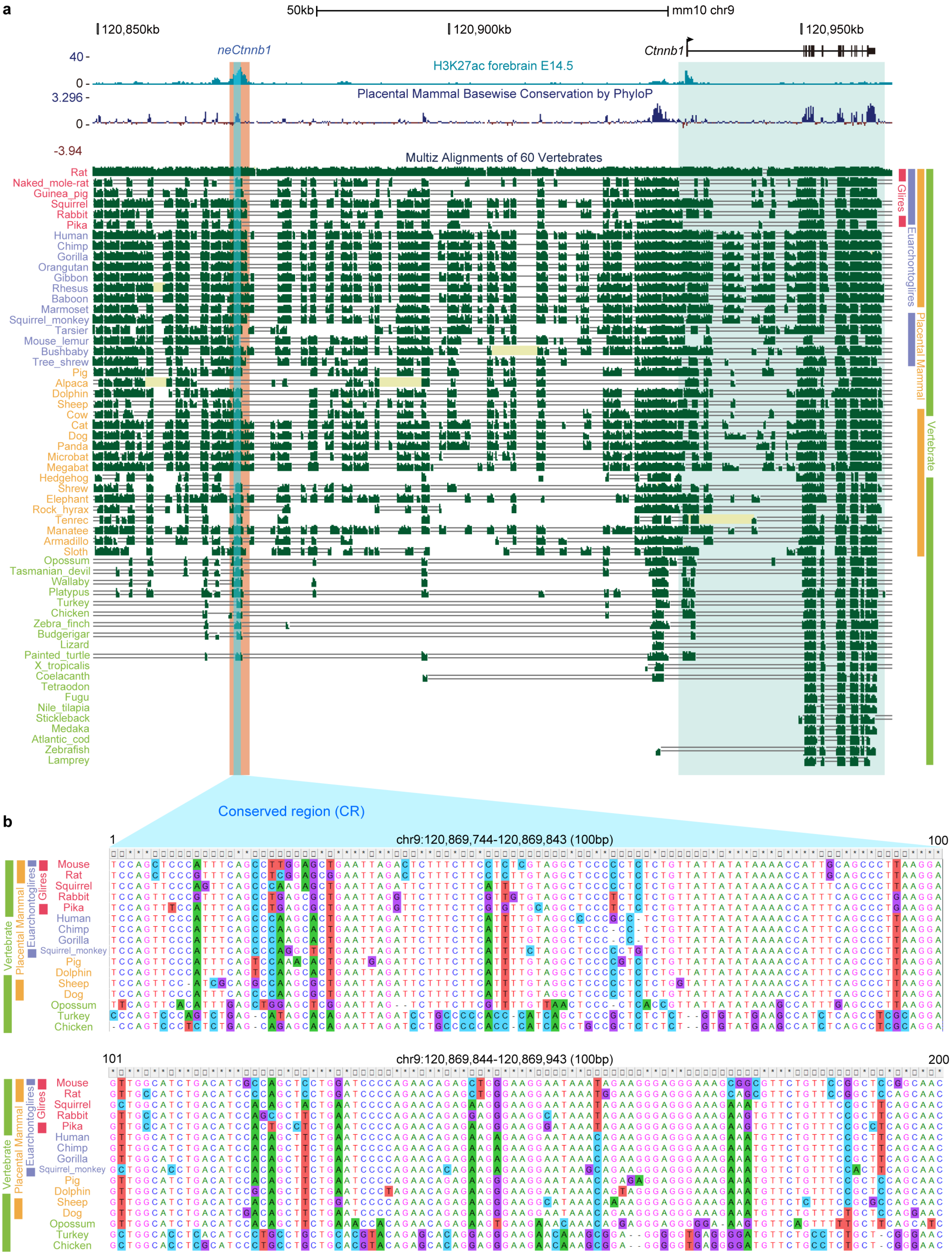

Supplementary Figure S6. Sequence conservation of *neCtnnb1*.

a UCSC genome browser track depicts conservation of *neCtnnb1* among vertebrates. **b** Multiple sequence alignment of the core conserved region of *neCtnnb1* among selected vertebrates. Data were extracted from the UCSC genome browser.

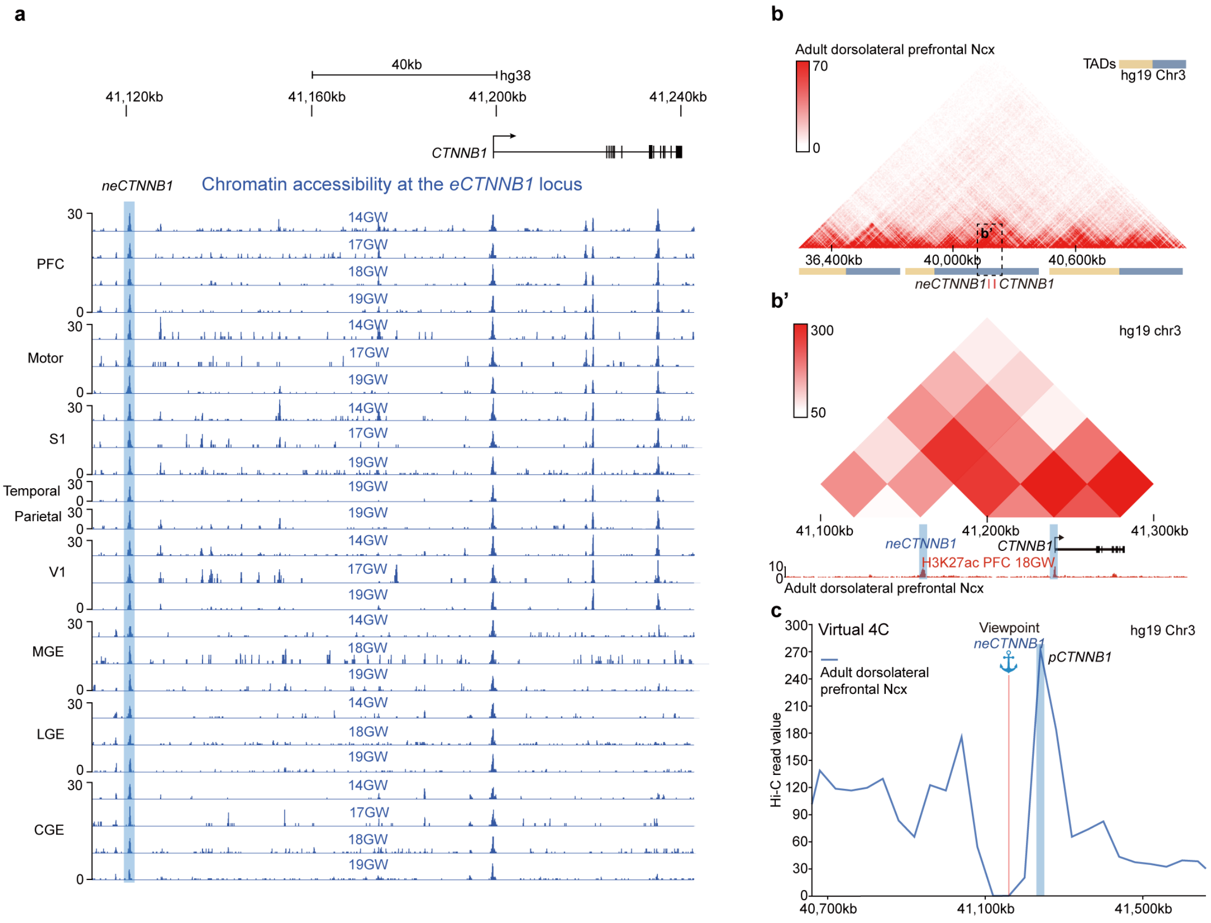

Supplementary Figure S7. *neCTNNB1* interacts with *pCTNNB1* in developing human brains.

a Schematic representation of ATAC-seq data upstream of human *CTNNB1* gene and the location of the putative enhancer *neCTNNB1* (blue shading). **b** The Hi-C data of adult dorsolateral prefrontal Ncx were obtained from the 3D genome browser. The boundaries of TADs and locations of *neCTNNB1* and *CTNNB1* are indicated below. **b**' Regions magnified from (**b**). *neCTNNB1* interacts with *pCTNNB1* in adult dorsolateral prefrontal Ncx. **c** Virtual 4C of adult dorsolateral prefrontal Ncx revealing interactions between *neCTNNB1* and *pCTNNB1*. PFC, dorsal lateral prefrontal cortex; Motor, motor cortex; S1, primary somatosensory cortex; Temporal, temporal cortex; Parietal, parietal cortex, V1, primary visual cortex; MGE, LGE, CGE, medial, lateral, and caudal ganglionic eminences.

Table S1. Summary of data used in this study.

Source name	Organism	Age	Library strategy	Antibody	Serial number
Prefrontal cortex	Homo sapiens	14gw	ATAC-seq	/	GSE149268
Prefrontal cortex	Homo sapiens	17gw	ATAC-seq	/	GSE149268
Prefrontal cortex	Homo sapiens	18gw	ATAC-seq	/	GSE149268
Prefrontal cortex	Homo sapiens	19gw	ATAC-seq	/	GSE149268
Motor cortex	Homo sapiens	14gw	ATAC-seq	/	GSE149268
Motor cortex	Homo sapiens	17gw	ATAC-seq	/	GSE149268
Motor cortex	Homo sapiens	19gw	ATAC-seq	/	GSE149268
Somatosensory cortex	Homo sapiens	14gw	ATAC-seq	/	GSE149268
Somatosensory cortex	Homo sapiens	17gw	ATAC-seq	/	GSE149268
Somatosensory cortex	Homo sapiens	19gw	ATAC-seq	/	GSE149268
Temporal cortex	Homo sapiens	19gw	ATAC-seq	/	GSE149268
Parietal cortex	Homo sapiens	19gw	ATAC-seq	/	GSE149268
Visual cortex	Homo sapiens	14gw	ATAC-seq	/	GSE149268
Visual cortex	Homo sapiens	17gw	ATAC-seq	/	GSE149268
Visual cortex	Homo sapiens	19gw	ATAC-seq	/	GSE149268
Medial ganglionic eminence	Homo sapiens	14gw	ATAC-seq	/	GSE149268
Medial ganglionic eminence	Homo sapiens	18gw	ATAC-seq	/	GSE149268
Medial ganglionic eminence	Homo sapiens	19gw	ATAC-seq	/	GSE149268
Lateral ganglionic eminence	Homo sapiens	14gw	ATAC-seq	/	GSE149268
Lateral ganglionic eminence	Homo sapiens	18gw	ATAC-seq	/	GSE149268
Lateral ganglionic eminence	Homo sapiens	19gw	ATAC-seq	/	GSE149268
Caudal ganglionic eminence	Homo sapiens	14gw	ATAC-seq	/	GSE149268
Caudal ganglionic eminence	Homo sapiens	17gw	ATAC-seq	/	GSE149268
Caudal ganglionic eminence	Homo sapiens	18gw	ATAC-seq	/	GSE149268
Caudal ganglionic eminence	Homo sapiens	19gw	ATAC-seq	/	GSE149268
Prefrontal cortex	Homo sapiens	15gw	ChIP-seq	H3K27ac	GSE149268
Prefrontal cortex	Homo sapiens	17gw	ChIP-seq	H3K27ac	GSE149268
Prefrontal cortex	Homo sapiens	18gw	ChIP-seq	H3K27ac	GSE149268
Prefrontal cortex	Homo sapiens	15gw	ChIP-seq	H3K4me1	GSE149268
Forebrain	Mus musculus	E11.5	ChIP-seq	H3K27ac	ENCSR275KPI
Forebrain	Mus musculus	E15.5	ChIP-seq	H3K27ac	ENCSR691NQH
Forebrain	Mus musculus	P0	ChIP-seq	H3K27ac	ENCSR094TTT
Forebrain	Mus musculus	E11.5	DNase-seq	/	ENCSR014SFF
Forebrain	Mus musculus	E14.5	DNase-seq	/	ENCSR337EDG
Forebrain	Mus musculus	P0	DNase-seq	/	ENCSR791AJY
Forebrain	Mus musculus	E11.5	ChIP-seq	H3K4me1	ENCSR975QSF
Forebrain	Mus musculus	E15.5	ChIP-seq	H3K4me1	ENCSR875KRK
Forebrain	Mus musculus	P0	ChIP-seq	H3K4me1	ENCSR465PLB

Forebrain	Mus musculus	E11.5	ChIP-seq	H3K4me3	ENCSR739DVM
Forebrain	Mus musculus	E15.5	ChIP-seq	H3K4me3	ENCSR022KDE
Forebrain	Mus musculus	P0	ChIP-seq	H3K4me3	ENCSR258YWW
Forebrain	Mus musculus	E11.5	ChIP-seq	H3K36me3	ENCSR066LZB
Forebrain	Mus musculus	E15.5	ChIP-seq	H3K36me3	ENCSR437SFX
Forebrain	Mus musculus	P0	ChIP-seq	H3K36me3	ENCSR069TDC
Midbrain	Mus musculus	E11.5	ChIP-seq	H3K27ac	ENCSR088UKA
Midbrain	Mus musculus	E15.5	ChIP-seq	H3K27ac	ENCSR428GHF
Midbrain	Mus musculus	P0	ChIP-seq	H3K27ac	ENCSR672ZXY
Midbrain	Mus musculus	E11.5	DNase-seq	/	ENCSR292QBA
Midbrain	Mus musculus	E14.5	DNase-seq	/	ENCSR367FCW
Midbrain	Mus musculus	P0	DNase-seq	/	ENCSR767AJS
Midbrain	Mus musculus	E11.5	ChIP-seq	H3K4me1	ENCSR450ITF
Midbrain	Mus musculus	E15.5	ChIP-seq	H3K4me1	ENCSR449EUZ
Midbrain	Mus musculus	P0	ChIP-seq	H3K4me1	ENCSR391WSS
Midbrain	Mus musculus	E11.5	ChIP-seq	H3K4me3	ENCSR283RFW
Midbrain	Mus musculus	E15.5	ChIP-seq	H3K4me3	ENCSR486MHP
Midbrain	Mus musculus	P0	ChIP-seq	H3K4me3	ENCSR427ZJU
Midbrain	Mus musculus	E11.5	ChIP-seq	H3K36me3	NCSR535NVF
Midbrain	Mus musculus	E15.5	ChIP-seq	H3K36me3	ENCSR487RAU
Midbrain	Mus musculus	P0	ChIP-seq	H3K36me3	ENCSR951UWY
Hindbrain	Mus musculus	E11.5	ChIP-seq	H3K27ac	ENCSR129LAP
Hindbrain	Mus musculus	E15.5	ChIP-seq	H3K27ac	ENCSR066XFL
Hindbrain	Mus musculus	P0	ChIP-seq	H3K27ac	ENCSR332JYZ
Hindbrain	Mus musculus	E11.5	DNase-seq	/	ENCSR358ESL
Hindbrain	Mus musculus	E14.5	DNase-seq	/	ENCSR179PIH
Hindbrain	Mus musculus	P0	DNase-seq	/	ENCSR469VGZ
Hindbrain	Mus musculus	E11.5	ChIP-seq	H3K4me1	ENCSR695FPP
Hindbrain	Mus musculus	E15.5	ChIP-seq	H3K4me1	ENCSR921ILW
Hindbrain	Mus musculus	P0	ChIP-seq	H3K4me1	ENCSR617VBE
Hindbrain	Mus musculus	E11.5	ChIP-seq	H3K4me3	ENCSR928CYU
Hindbrain	Mus musculus	E15.5	ChIP-seq	H3K4me3	ENCSR335TVR
Hindbrain	Mus musculus	P0	ChIP-seq	H3K4me3	ENCSR472YGQ
Hindbrain	Mus musculus	E11.5	ChIP-seq	H3K36me3	ENCSR175QZX
Hindbrain	Mus musculus	E15.5	ChIP-seq	H3K36me3	ENCSR809NWL
Hindbrain	Mus musculus	P0	ChIP-seq	H3K36me3	ENCSR458PAO
Forebrain	Mus musculus	E12.5	ChIP-seq	P300	GSE88789
Facial prominence	Mus musculus	E15.5	ChIP-seq	H3K27ac	ENCSR382DRK
Heart	Mus musculus	E15.5	ChIP-seq	H3K27ac	ENCSR574VME
Intestine	Mus musculus	E15.5	ChIP-seq	H3K27ac	ENCSR599GVS

Kidney	Mus musculus	E15.5	ChIP-seq	H3K27ac	ENCSR711SVB
Limb	Mus musculus	E15.5	ChIP-seq	H3K27ac	ENCSR988BRP
Liver	Mus musculus	E15.5	ChIP-seq	H3K27ac	ENCSR479LFP
Lung	Mus musculus	E15.5	ChIP-seq	H3K27ac	ENCSR895BMP
Neural tube	Mus musculus	E15.5	ChIP-seq	H3K27ac	ENCSR241BSK
Stomach	Mus musculus	E15.5	ChIP-seq	H3K27ac	ENCSR929SEW
Cortical plate	Mus musculus	8 weeks	ChIP-seq	H3K27ac	ENCSR000CDD
Cortex	Mus musculus	E14.5	ChIP-seq	TBR2	GSE63621
Cortex	Mus musculus	E14.5	ChIP-seq	NEUROD2	GSE67539
Cortex	Mus musculus	E14.5	ChIP-seq	NEUROG2	GSE63621
Embryonic neural stem cells	Mus musculus	E14.5	ChIP-seq	FOXP1	GSE101632
Cortex	Mus musculus	E15	ChIP-seq	SATB2	GSE68910
Cortex	Mus musculus	E12.5	ChIP-seq	SMAD3	GSE36673
ES-derived NPCs	Mus musculus	/	ChIP-seq	SOX2	GSE35496
ES-derived NPCs	Mus musculus	/	ChIP-seq	YY1	GSE25197
AtT-20 cells	Mus musculus	/	ChIP-seq	ASH2	GSE87180
Embryonic Stem Cells	Mus musculus	E14	ChIP-seq	GFP	GSE52071
B cells	Mus musculus	6-14 weeks	ChIP-seq	ASH2	GSE20852
in vitro differentiated NPC	Mus musculus	/	Hi-C	/	GSE96107
Neuron	Mus musculus	E14.5	Hi-C	/	GSE96107
H1 Neuronal Progenitor cells	Homo sapiens	/	Hi-C	/	GSE52457
H1 embryonic stem cells	Homo sapiens	/	Hi-C	/	GSE52457
H1 Mesendoderm cells	Homo sapiens	/	Hi-C	/	GSE52457
H1 Trophectoderm cells	Homo sapiens	/	Hi-C	/	GSE52457
H1 Mesenchymal stem cells	Homo sapiens	/	Hi-C	/	GSE52457
Dorsolateral Prefrontal Cortex	Homo sapiens	Adult	Hi-C	/	GSE87112

Table S2. Primers used in this study.

Primers	Sequence (5'-3')	Description
sg1	gtgagaagcaagagctcact	used for CRISPRa
sg2	ggaggcagctgtgggcctga	used for CRISPRa and FLAG-dCas9-ChIP to *neCtnnb1*
sg1	cctgggcagagcttaagcag	used for CRISPRi
sg2	ggaagctgccatgatgcaa	used for CRISPRi
sg1	ggcgcacgcgcagagcgact	used for FLAG-dCas9-ChIP to *pCtnnb1*
sg1	atacagcatgagagcaccgt	used for screen functional region of *neCtnnb1*
sg2	gacactttcgtcgctgctcc	used for screen functional region of *neCtnnb1*
sg3	ggggtctcctagacatgtag	used for screen functional region of *neCtnnb1*
sg4	ggaggcagctgtgggcctga	used for screen functional region of *neCtnnb1*
sg5	gacactatagcttaccctgc	used for screen functional region of *neCtnnb1*
sg6	taactgtgttgtccgcccag	used for screen functional region of *neCtnnb1*
sg7	atgtcacataggatttacgg	used for screen functional region of *neCtnnb1*
sg8	actctacatggagtgccgtg	used for screen functional region of *neCtnnb1*
sg9	gtggggactagccctgcttg	used for screen functional region of *neCtnnb1*
sg10	gaagggttaattccgaacct	used for screen functional region of *neCtnnb1*
*neCtnnb1*_3C_1	cctgactgaagcaggcatcg	used for 3C
*neCtnnb1*_3C_2	gcagggagttacccggtagaa	used for 3C
*neCtnnb1*_3C_3	agactctttcttcctctcgtaggct	used for 3C
*neCtnnb1*_3C_4	atgatcagtgagagcctgaaatgaa	used for 3C
*neCtnnb1*_3C_5	actcatctctctctgcttcccaatat	used for 3C
*neCtnnb1*_3C_6	tggcacttcaagtctcttccagg	used for 3C
*pCtnnb1*_3C	gccactcctggtgttggagg	used for 3C
Probe_*Ctnnb1*_F	cacgactagttcagctgcttgt	used for ISH
Probe_*Ctnnb1*_R	tccacacatgaacatctccttc	used for ISH
*neCtnnb1*_ChIP_F	accgtcagagagttaacagc	used for ChIP-qPCR
*neCtnnb1*_ChIP_R	actccccctctggtgggtact	used for ChIP-qPCR
*pCtnnb1*_ChIP_F	ggcccgctctcgattccttt	used for ChIP-qPCR
*pCtnnb1*_ChIP_R	actggcggggactactttcc	used for ChIP-qPCR
*Ctnnb1*_Ex3_F	atggagccggacagaaaagc	used for ChIP-qPCR

*Ctnnb1*_Ex3_R	cttgccactcagggaagga	used for ChIP-qPCR
*neCtnnb1*_ctrl_F	ctgcttaagctctgcccagg	used for ChIP-qPCR
*neCtnnb1*_ctrl_R	ttgacatcatggcagcttcc	used for ChIP-qPCR
hDesert_F	gggagatggagaaaggtggaaggtt	used for ChIP-qPCR
hDesert_R	cccaaggccataaaatatgagctggg	used for ChIP-qPCR
pCTNNB1-ChIP-F	gagaggtgggatccaccatcc	used for ChIP-qPCR
pCTNNB1-ChIP-R	tcgccgaccttgtggtctgtg	used for ChIP-qPCR
neCtnnb1-ChIP-F	gcgggcttcttatccccatctg	used for ChIP-qPCR
neCtnnb1-ChIP-R	tgatgtggcaccttcgggacc	used for ChIP-qPCR
Ash2l_sh1	ccttaggctatgataagttta	used for knockdown *Ash2l*
Ash2l_sh2	cctgtgtctgtgtgttccaaa	used for knockdown *Ash2l*
ASH2L_sh1	gtgacttgttatcctactata	used for knockdown *ASH2L*
ASH2L_sh2	cctgcttgtatgaacgggttt	used for knockdown *ASH2L*
*Ash2l*_F	cctactttctccggaagcaag	used for RT-qPCR
*Ash2l*_R	gacaatgttattgggccaagtc	used for RT-qPCR
*ASH2L*_F	agaatggccgacagttggg	used for RT-qPCR
*ASH2L*_R	ccttcaagtttgcttgcttcc	used for RT-qPCR
*Ctnnb1*_F	atggagccggacagaaaagc	used for RT-qPCR
*Ctnnb1*_R	cttgccactcagggaagga	used for RT-qPCR
*CTNNB1*_F	aaagcggctgttagtcactgg	used for RT-qPCR
*CTNNB1*_R	cgagtcattgcatactgtccat	used for RT-qPCR
*Satb2*_F	gccgtgggaggtttgatgatt	used for RT-qPCR
*Satb2*_R	accaagacgaactcagcgtg	used for RT-qPCR
*Neurod2*_F	aagccagtgtctcttcgtgg	used for RT-qPCR
*Neurod2*_R	gccttggtcatcttgcgttt	used for RT-qPCR
*Sox2*_F	gcggagtggaaacttttgtcc	used for RT-qPCR
*Sox2*_R	cgggaagcgtgtacttatcctt	used for RT-qPCR
*Yy1*_F	cagtggttgaagagcagatcat	used for RT-qPCR
*Yy1*_R	agggagtttcttgcctgtcat	used for RT-qPCR
*Gapdh*_F	aggtcggtgtgaacggatttg	used for RT-qPCR
*Gapdh*_R	tgtagaccatgtagttgaggtca	used for RT-qPCR
Ctnnb1$^{lox(ex3)}$-F	ggtagtggtccctgcccttgacac	used for validation of *Ctnnb1*$^{lox(ex3)}$ mice
Ctnnb1$^{lox(ex3)}$-AS5	acgtctggcaagttccgcgtcatcc	used for validation of *Ctnnb1*$^{lox(ex3)}$ mice
Ctnnb1$^{lox(ex3)}$-P85	ctaagcttggctggacgtaaactc	used for validation of *Ctnnb1*$^{lox(ex3)}$ mice
Ctnnb1^{KO} -WT-F	ccctgcccctgcatatagcatttga	used for validation of *Ctnnb1*^{KO} mice
Ctnnb1^{KO} -WT-R	ccccacctgtgatgctttgatgtct	used for validation of *Ctnnb1*^{KO} mice
Ctnnb1^{KO} -Mut-R	atgctgtgtgagtgaccctactcct	used for validation of *Ctnnb1*^{KO} mice
LacZ F	atcctctgcatggtcaggtc	used for validation of neCtnnb1-LacZ-iCre and BAT-Gal mice
LacZ R	cgtggcctgattcattcc	used for validation of neCtnnb1-LacZ-iCre and BAT-Gal mice
